# Fine-Tuning of Energy Levels Regulates *SUC2* via a SNF1-Dependent Feedback Loop

**DOI:** 10.3389/fphys.2020.00954

**Published:** 2020-08-14

**Authors:** Sebastian Persson, Niek Welkenhuysen, Sviatlana Shashkova, Marija Cvijovic

**Affiliations:** ^1^Department of Mathematical Sciences, University of Gothenburg, Gothenburg, Sweden; ^2^Department of Mathematical Sciences, Chalmers University of Technology, Gothenburg, Sweden; ^3^Department of Microbiology and Immunology, Institute of Biomedicine, Sahlgrenska Academy, University of Gothenburg, Gothenburg, Sweden

**Keywords:** SNF1, feedback, single-cell, nutrient signaling, dynamic modeling, NLME, STS

## Abstract

Nutrient sensing pathways are playing an important role in cellular response to different energy levels. In budding yeast, *Saccharomyces cerevisiae*, the sucrose non-fermenting protein kinase complex SNF1 is a master regulator of energy homeostasis. It is affected by multiple inputs, among which energy levels is the most prominent. Cells which are exposed to a switch in carbon source availability display a change in the gene expression machinery. It has been shown that the magnitude of the change varies from cell to cell. In a glucose rich environment Snf1/Mig1 pathway represses the expression of its downstream target, such as *SUC2*. However, upon glucose depletion SNF1 is activated which leads to an increase in *SUC2* expression. Our single cell experiments indicate that upon starvation, gene expression pattern of *SUC2* shows rapid increase followed by a decrease to initial state with high cell-to-cell variability. The mechanism behind this behavior is currently unknown. In this work we study the long-term behavior of the Snf1/Mig1 pathway upon glucose starvation with a microfluidics and non-linear mixed effect modeling approach. We show a negative feedback mechanism, involving Snf1 and Reg1, which reduces *SUC2* expression after the initial strong activation. Snf1 kinase activity plays a key role in this feedback mechanism. Our systems biology approach proposes a negative feedback mechanism that works through the SNF1 complex and is controlled by energy levels. We further show that Reg1 likely is involved in the negative feedback mechanism.

## 1. Introduction

Nutrients play a key role in cell survival and well-being by serving as energy sources, cellular building blocks and as triggers for a multitude of signaling pathways. A number of nutrient-controlled signaling pathways has been extensively studied and the crosstalk between them has been elucidated (Shashkova et al., 2015). In the budding yeast, *Saccharomyces cerevisiae*, nutrient controlled pathways distinguish between preferred and alternative nitrogen and carbon sources, and alters the homeostasis to adjust to the extracellular conditions. The Snf1 protein kinase, the yeast orthologue of the mammalian AMP-activated protein kinase (AMPK), regulates energy balance and plays the main role in yeast adaptation to glucose limitation (Carlson et al., 1981; Celenza and Carlson, 1986) via controlling genes required for utilization of non-glucose carbon sources (Treitel et al., 1998). It works in a complex, named SNF1, which is composed of the catalytic subunit Snf4 and one of the three alternative stabilizing subunits Gal83, Sip1, or Sip2 (Jiang and Carlson, 1996; Schmidt and McCartney, 2000). Under glucose depleted conditions, AMPK/Snf1 is activated by three upstream kinases, Tos3, Elm1 and Sak1 (Hong et al., 2003; Nath et al., 2003; Elbing et al., 2006; Rubenstein et al., 2008), which leads to phosphorylation of various transcription factors to facilitate cellular response (Ghillebert et al., 2011). Glucose presence makes the Snf1 activation loop accessible for protein phosphatases (Rubenstein et al., 2008). The Glc7-Reg1 phosphatase is the main negative regulator of Snf1, with a contribution of Sit4 and Ptc1 phosphatases (Rubenstein et al., 2008; Ruiz et al., 2012). At the same time, the assembly and functionality of the Glc7-Reg1 phosphatases depends on Snf1 during poor glucose conditions; active Snf1 phosphorylates Reg1 which prevents its association with the Glc7 subunit to form a functional phosphatase (Sanz et al., 2000). The activation of the SNF1 complex has been shown to correlate with a high ADP/ATP ratio (Rubenstein et al., 2008; Chandrashekarappa et al., 2013). Furthermore, ADP binds to the regulatory subunit of the SNF1 complex resulting in protection of Thr210 from dephosphorylation (Mayer et al., 2011). Overall, this suggests that the SNF1 complex is regulated by intracellular energy levels.

Genes essential for metabolism of maltose (*MAL*), galactose (*GAL*), and sucrose (*SUC2*) are regulated by the transcriptional repressor Mig1, where *SUC2* is one of the most studied (Lutfiyya et al., 1998; Carlson, 1999). Expression of Mig1 target genes is released upon glucose limitation, when Mig1 becomes phosphorylated by Snf1 and relocates to the cytoplasm. In glucose-rich extracellular conditions, the Reg1-Glc7 phosphatase dephosphorylates Mig1 in a glucose-dependent manner, however, another glucose-independent mechanism has been reported to participate in Mig1 dephopshorylation (Shashkova et al., 2017). When dephopshorylated, Mig1 relocates to the nucleus (Treitel and Carlson, 1995; Wu and Trumbly, 1998) where it recruits the Ssn6-Tup1 global co-repressor complex to repress genes (Lutfiyya et al., 1998; Smith et al., 1999; Ahuatzi et al., 2007).

**Figure 1:**
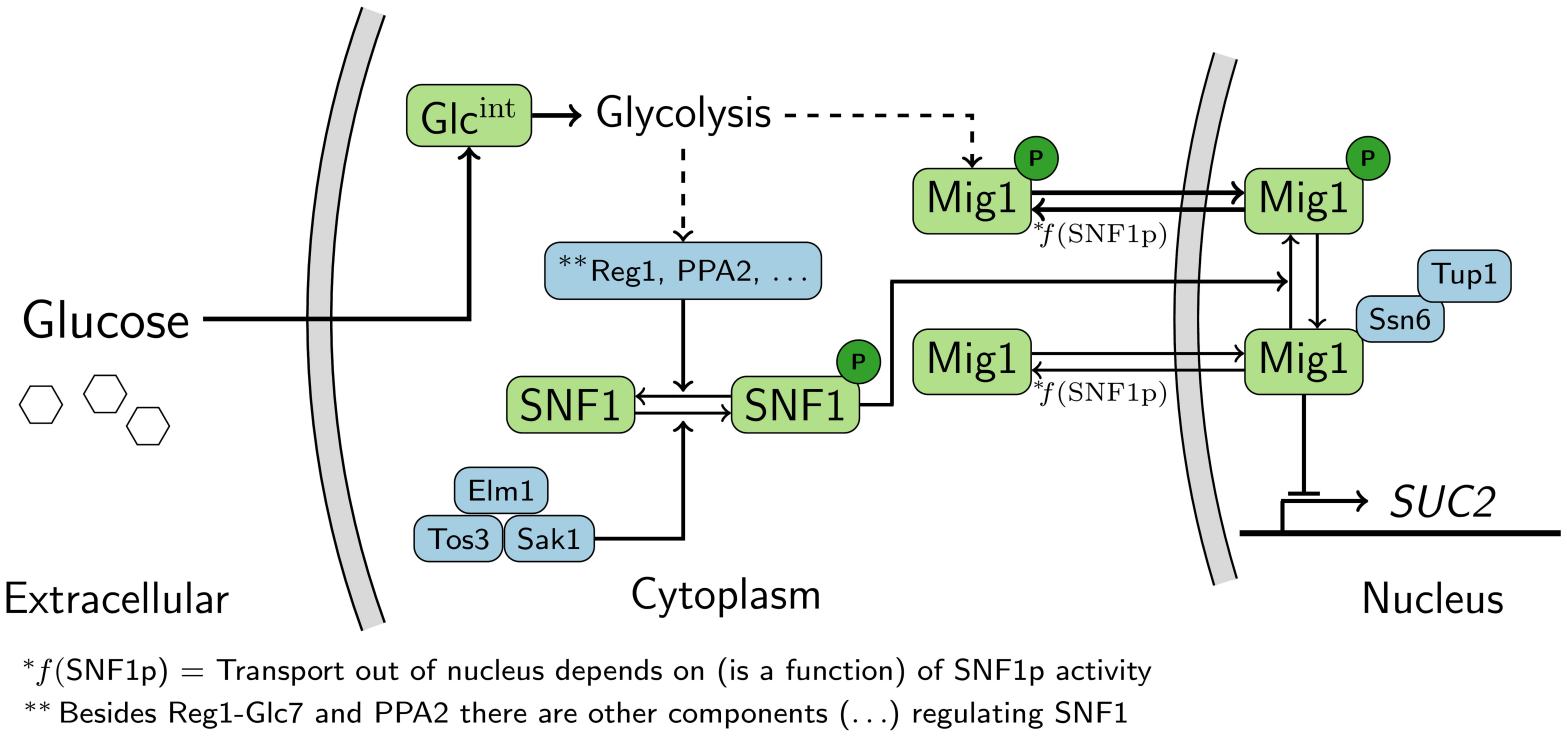
Schematic representation of the Snf1/Mig1 pathway. Snf1 is constitutively phosphorylated by the upstream kinases. When preferred carbon-sources are limited Snf1 phosphorylates Mig1, which results in the cytoplasmic localization of Mig1. When preferred carbon sources are available Snf1 and Mig1 are dephosphorylated through an unknown mechanism originating from glycolysis. Mig1 localizes to the nucleus and there it represses genes which are required for the utilization of non-fermentable carbon-sources. Dashed arrows represent signals that are transmitted via unknown mechanisms.

Despite the fact the Snf1/Mig1 pathway (Figure 1) has been extensively studied, little is known about how its response to glucose starvation is maintained over time. Thus, it is still unclear how the gene expression response to altered energy levels is fine-tuned, for example, the dynamic regulation of the pathway target genes in the long run. Also, the majority of work on the Snf1/Mig1 pathway has been performed on cell cultures representing the average behavior of the population. When the aim is a cellular level mechanistic understanding of a pathway, such as the Snf1/Mig1 pathway, a single-cell level model is advantageous compared to a population average model. This is due to the fact that population based models do not account for intrinsic heterogeneity within the population (Shashkova and Leake, 2017). Hence, a population based model might overlook dynamic single-cell features, such as oscillations (Cohen-Saidon et al., 2009).

Systems biology approaches exploiting single-cell techniques to study the cellular mechanism of the Snf1/Mig1 pathway (Bendrioua et al., 2014; Almquist et al., 2015; Welkenhuysen et al., 2017) have previously been employed. However, these have focused on the short-term localization of Mig1 as measurement for the Snf1/Mig1 pathway activity. Long time-lapse observation of pathways with microfluidics device provide high-quality observation rich data. In combination with mathematical modeling this data can be used to understand complex behavior of cellular pathways. The abundance of species in Snf1/Mig1 pathway (Shashkova et al., 2015) together with previous modeling studies (Almquist et al., 2015), suggest that the cell-to-cell variability in Snf1/Mig1 pathway mostly arises from differences in protein levels (extrinsic noise). Therefore, a suitable modeling framework to study single-cell activity of the Snf1/Mig1 pathway is non-linear mixed effects modeling (NLME) (Almquist et al., 2015; Llamosi et al., 2016).

In this work we study the long-term behavior of the Snf1/Mig1 pathway upon glucose starvation with a microfluidics and NLME modeling approach. We employ a single-cell microfluidics technique to capture the long-term behavior of a large set of cells after the nutrients conditions in the cell environment have changed. We further examined activity levels of the invertase enzyme as a readout of the *SUC2* expression to investigate the effect of Snf1 and Reg1 on target genes. Moreover, we utilize a standard fluorescence microscopy approach on cells subjected to a pharmacological inhibitor to show the crucial role of the Snf1 kinase activity in regulation of Mig1 localization that defines its function on target promoters. We show that a feedback mechanism reduces *SUC2* expression after the initial strong activation. Our modeling approach proposes a negative feedback mechanism that works through the SNF1 complex and is controlled by energy levels. We further show that Reg1 is involved in the negative feedback mechanism.

## 2. Materials and Methods

### 2.1. Yeast Strains and Growth Conditions

Standard YPD (10 g/l yeast extract, 20 g/l bacto-peptone, glucose according to experimental needs) and YNB [1.7 g/l yeast nitrogen base without amino acids, without (NH_4_)_2_SO_4_, 5 g/l (NH_4_)_2_SO_4_, supplemented with glucose and amino acids according to nutritional requirements] media were used for yeast cells growth and transformants selection.

To delete the *SNF1* gene, the *LEU2* fragment from YDp-L plasmid (Berben et al., 1991) flanked on its 5′- and 3′-termini with 50 bp up- and downstream of *SNF1*, respectively, was amplified by PCR. Strain YSH2348 was transformed directly with the PCR reaction mix by standard lithium acetate protocol (Gietz and Schiestl, 2007) and placed on YNB leucin-deficient agar plates supplemented with 4% glucose. Successful transformants were verified by confirmation PCR.

### 2.2. Invertase Assay

Pre-grown cells were inoculated into 50 ml of fresh YPD medium with 4% glucose and grown until mid-log phase. A half of a culture then was harvested by rapid centrifugation and freezing in liquid nitrogen, the other half was washed with water, suspended in a fresh YPD medium with 0.2% glucose, incubated at 30°C, 180 rpm for following 4 h and harvested as above. Yeast cells were mechanically disrupted by glass beads in crude extraction buffer [50 mM imidazole, 100 mM KCl, 10 mM MgCl_2_, 0.1 mM EDTA, 1 × protease inhibitor cocktail (Roche)], and the lysates we obtained by collecting supernatants after centrifugation for 10 min at 10,000 rpm. The total amount of protein was quantified by RC DC protein quantification kit (Bio-Rad). Protein extracts were mixed with acetate buffer (0.3 M CH_3_COOH, 0.2 M CH_3_COOK), and the reaction was initiated by adding 500 mM sucrose solution (in 0.1 M CH_3_COOK). After 10 min the reaction mix was added to 0.1 M KPO_4_ pH 6.5 containing peroxidase, glucose oxidase, and O-dianisidine, and incubated at 30°C water bath for 15 min. The reaction was stopped by adding 6M HCl, and the OD at 540 nm was measured. One unit of invertase activity is the amount of enzyme that produces 1 nmol of glucose per minute at pH 6.5 at 30°C.

### 2.3. Epifluorescence Microscopy

Pre-grown cells carrying *pSNF1-TAP* or its' analog-sensitive version, *pSNF1-I132G-TAP*, were cultivated in YNB medium with uracil-deficient amino acid supplement with 0.2% glucose for 1 h. To block Snf1 phosphorylation, 2 μM ATP-competitive kinase inhibitor, 1NM-PP1 (Cayman), was added to the cell cultures for 5 min at room temperature. For the wide field fluorescence microscopy cells were imaged using an ApoTome camera and a Zeiss Axiovert 200M microscope (Carl Zeiss MicroImaging). Fluorescence images were acquired by using separate filter sets 38HE and 43HE for GFP and mCherry excitation, respectively.

### 2.4. Time-Lapse Microscopy and Cell Tracking

The yeast cells (W303, HXK1p-Citrine-ACT1t, Table S3) were grown overnight and injected with a syringe in a two-channel Y-formed microfluidics poly-dimethylsiloxane (PDMS) system and allowed to sediment in the main channel. For the switch fresh CSM media was supplied to the cells through the other channel. The experimental setup is further described in Welkenhuysen et al. (2018). Imaging was performed on a Leica DMi8 inverted fluorescence microscope (Leica microsystems). The microscope was equipped with a HCX PL APO 40 × /1.30 oil objective (Leica microsystems), Lumencor SOLA SE (Lumencor) led light and Leica DFC9000 GT sCMOS camera (Leica microsystems). Cell growth was recorded at 1 frame in bright-field at 20 ms exposure, and YFP was observed with an excitation: 500/20, dichroic: 515 and emission: 535/30 filtercube at 150 ms exposure every 5 min. Analysis of fluorescence intensity was performed with the ImageJ distribution FIJI (Schindelin et al., 2012).

### 2.5. Feedback Cascade Model

The simple feedback cascade model (Figure 3A) is based on ODEs with the rate-equations formulated using mass action and Hill-kinetics (Equation 1). The model has three components: Snf1/Mig1 pathway inhibitory activity (*SNF*1*pat*), *SUC2* and a potential feedback cascade (*X*). The model investigates if a partial recovery in intracellular energy levels, that results in a recovery in *SNF*1*pat* activity (meaning the inactivation of Snf1 and activation of Mig1) (Mayer et al., 2011), might explain the observed *SUC2* expression. This is achieved by including the likely recovery in intracellular energy levels, which is an effect of genes activated upon a glucose drop and the decreased activity of *SNF*1*pat* (Hedbacker and Carlson, 2008), via the production of *X* (Equation 1c). Furthermore, as a partial recovery in energy levels should result in increased activity of the energy regulated *SNF*1*pat*, *X* promotes production of *SNF*1*pat* (Equation 1a). That is, *X* partially restores *SNF*1*pat* activity, which is a measure of the intracellular energy levels during high glucose conditions (i.e., high intracellular energy level).

(1a) $$\begin{array}{r} \frac{\text{d}SNF1pat\left(t\right)}{\text{d}t}=k_{\text{glc\_ex}}\left(t\right)-k_{2}SNF1pat\left(t\right)+k_{3}X\left(t-\tau_{\text{f}}\right) \end{array}$$

(1b) $$\begin{array}{r} \frac{\text{d}SUC2\left(t\right)}{\text{d}t}=\frac{k_{4}}{k_{5}+SNF1pat\left(t-\tau_{\text{m}}\right)}-k_{6}SUC2\left(t\right) \end{array}$$

(1c) $$\begin{array}{r} \frac{\text{d}X\left(t\right)}{\text{d}t}=H\left(t\right)\frac{k_{7}}{k_{8}+SNF1pat\left(t\right)}-k_{9}X\left(t\right) \end{array}$$

(1d) $$\begin{array}{r} \;SNF1pat\left(t\right)=SNF1pat_{t_{0}}\;\forall t\in \left[t_{0}-\tau_{\text{m}},t_{0}\right] \end{array}$$

(1e) $$\begin{array}{r} SUC2\left(t\right)=SUC2_{t_{0}},\;t=t_{0} \end{array}$$

(1f) $$\begin{array}{r} X\left(t\right)=0,\;\forall t\in \left[t_{0}-\tau_{\text{f}},t_{0}\right] \end{array}$$

**Figure 2:**
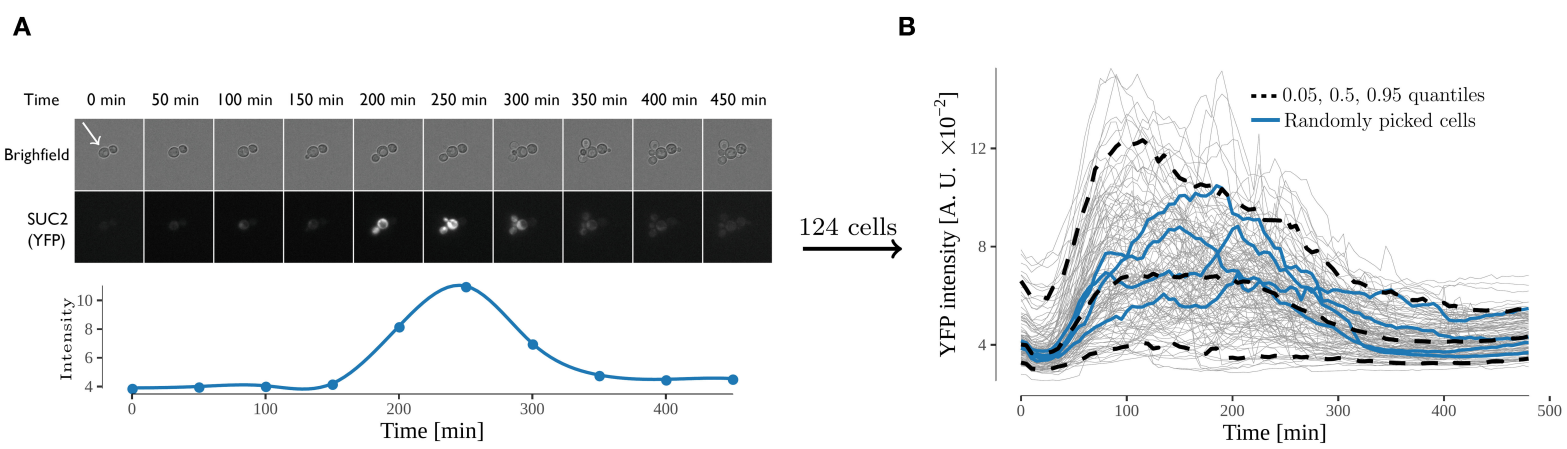
Expression from the *SUC2* promotor over time, expressed as fluorescence intensity of YFP divided by area. **(A)** time-lapse images of cells exposed to shift from high (4%) to low (0.1%) glucose. Both bright field and yellow fluorescence images are displayed. Below is the intensity over time of one cell (annotated by white arrow). The fluorescence intensity was observed for 480 min with an interval of 5 min between every image acquisition. **(B)** Fluorescence intensity over time of all analyzed cells. Each gray line represents the trace of one single cell, and the 0.05, 0.5, and 0.95 quantiles of the cells are represented with a dashed black line. Four randomly selected cells are represented with a blue line. 124 cells were analyzed in the experiment.

**Figure 3:**
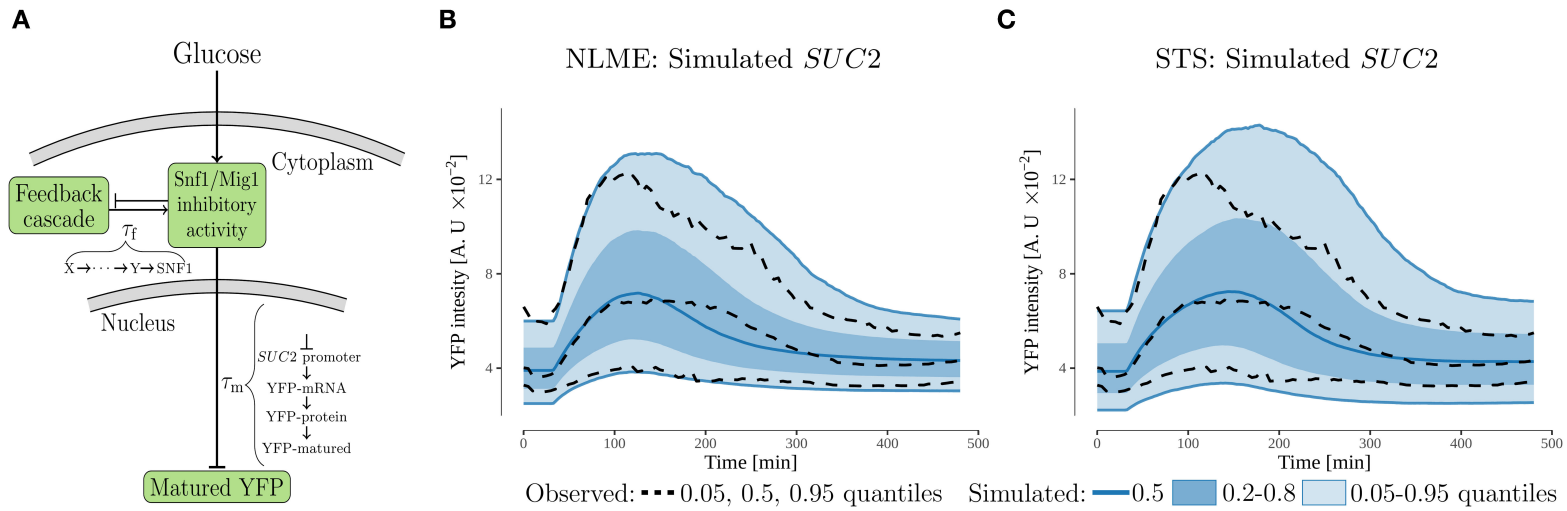
The feedback cascade model. **(A)** Schematic representation. The model proposes that the *SUC2* expression decreases during glucose starvation due to a feedback acting on the inhibitory activity of the Snf1/Mig1 pathway. A potential source of this feedback is partial recovery in the intracellular energy levels. The feedback-cascade, and maturation time of the promoter_*SUC*2_-YFP (measured output), were represented by time-delays, τ_*i*_. **(B,C)** Simulated and observed population behavior of the SUC2-intensity (note same scale on y-axis). The simulated data was generated by simulating 10,000 cells using the estimated parameter distributions for the NLME and STS approaches.

Besides standard model building procedures, three challenges had to be addressed when constructing the model. Firstly, the model aims to describe the *SUC2* expression. However, the *SUC2* expression is measured via matured YFP. Secondly, the feedback affecting the SNF1 pathway is likely a cascade of events. The fluorescence maturation and feedback cascade can be represented by adding multiple states to the model. However, to keep the model identifiable, time-delays τ_*i*_ were used instead (Figure 3A). As the data shows a variation in feedback response-time (Figure 2B), τ_f_ was estimated from the data. As the YFP maturation time has been reported to be 39±7 min (Gordon et al., 2007), and the data shows an increase in YFP at 32 min (Figure 2B), τ_m_ was fixated at 32 min. Thirdly, at time zero the model is at rich glucose conditions. At rich glucose conditions, the SNF1 pathway, and thus the model should be at steady state. Hence, the rate equations were forced to be zero at time zero, which yielded expressions for initial values, as functions of rate constants, that ensures an initial steady-state (Equation 2b). The glucose downshift which breaks the steady state, was included in the model by reducing glucose in-signal and activating the feedback mechanism close to time zero (Equation 2a). It should be noted that *X* initially in high glucose, is modeled to not interact with *SNF*1*pat* and thus has zero activity (Equations 1c and 2a). This is because the feedback is modeled to be dependent on the energy saving processes that are activated upon an external glucose drop (Hedbacker and Carlson, 2008). In addition to the information presented here, a more detailed description of each state and accompanying rate equations is presented in Table S1.

(2a) $$k_{\text{glc}\_\text{ex}}(t)\;=\{\begin{array}{l} k_{1}\;t<0.0483\\ k_{1}/40\;t\geq 0.0483 \end{array}\;,\;H(t)=\{\begin{array}{l} 0\;t<0.0483\\ 1\;t\geq 0.0483 \end{array}$$

(2b) $$SNF1pat_{t_{0}}=k_{1}/k_{2},\;SUC2_{t_{0}}=\frac{k_{4}}{(k_{5}+SNF1pat_{t_{0}})k_{6}}$$

When fitting the model to the *SUC2* data, all unknown parameters except two (*k*_5_ and *k*_8_) were estimated with a full random effects covariance matrix Ω. These two parameters were estimated without random effects to reduce the size of Ω, and consequently keeping the standard errors of the estimated parameters acceptable (Table S4). It should also be noted that fixing *k*_5_ and *k*_8_ does not, unrealistically, removes cell-to-cell variability in the reactions they govern. This is because *k*_4_ and *k*_7_ are assumed to vary between cells (Equations 1b and 1c). The unidentifiable Hill-coefficients (Equations 1b and 1c) where fixed to the smallest integer that produced a good fit (*n* = 1). In addition to the information presented here, inference details for each parameter is presented in Table S1.

### 2.6. Feedback Mediated Model

**Figure 4:**
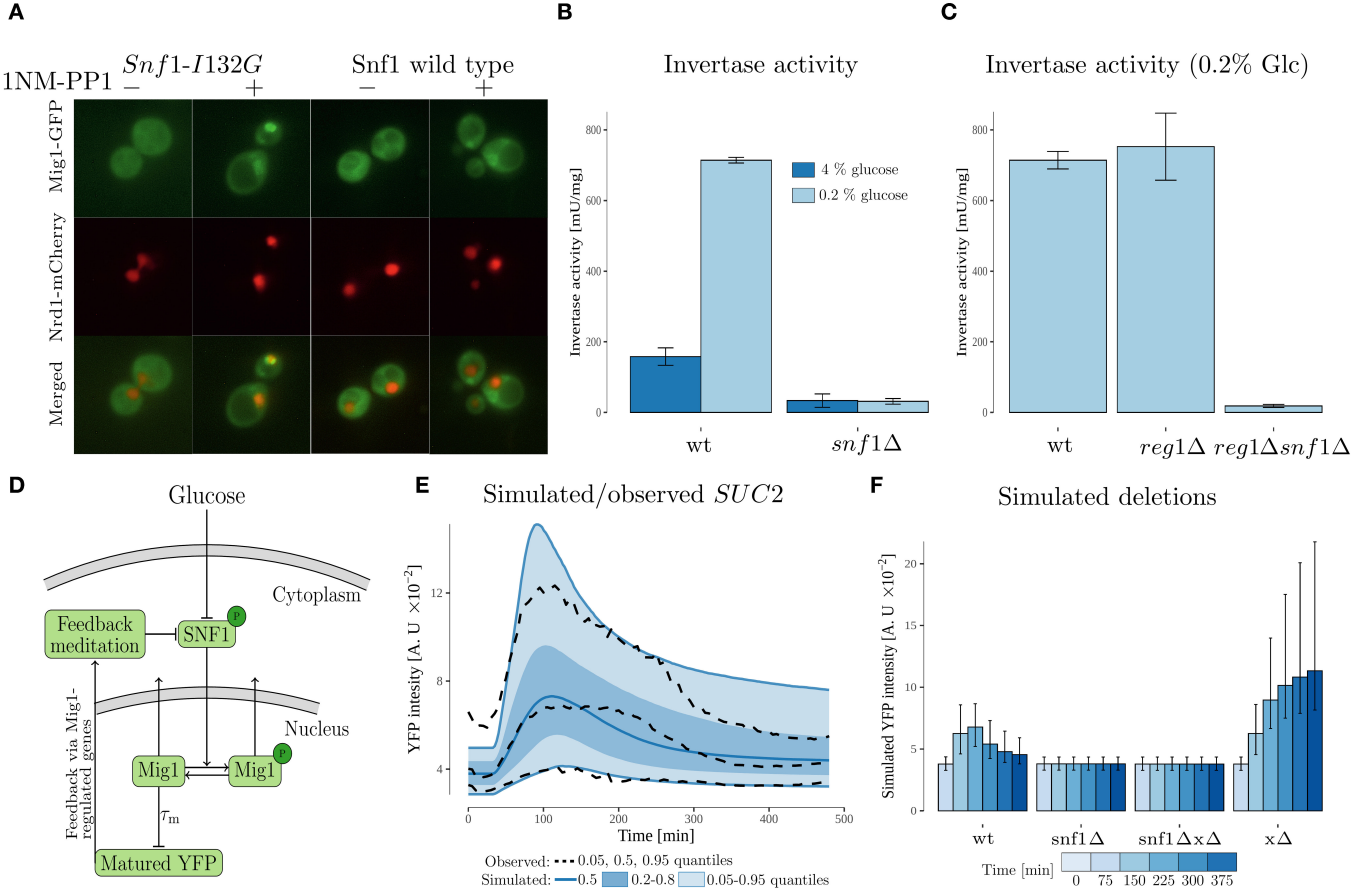
Experimental data and modeling suggests *SUC2* regulating feedback via SNF1 complex. **(A)** Cells with genomically integrated Mig1-GFP and Nrd1-mCherry fusions and expressing the wild type Snf1 or its analog-sensitive version, *Snf1-I132G*, were incubated in low (0.2%) glucose conditions with subsequent addition of 1NM-PP1 for 5 min prior to imaging. Images were enhanced using FiJi software for representation. **(B)** A read-out of invertase activity measured in cell lysates obtained from cultures pre-grown in 4% glucose medium, then incubated in 0.2% glucose conditions for 4 h. Error bars represent standard deviation. **(C)** A read-out of invertase activity measured in cell lysates obtained from cultures pre-grown in 4% glucose medium, then incubated in 0.2% glucose conditions for 4 h. Error bars represent standard deviation. **(D)** Feedback mediated model. The model, which is an extension of the cascade model (Figure 3), proposes that the *SUC2* expression decreases upon long term glucose starvation due to a feedback acting on the phosphorylated SNF1-complex (*SNF*1*p*). The maturation time of the promoter_*SUC*2_-YFP (measured output) was represented by a time-delay. The Mig1 nuclear export was represented by a *SNF*1*p* dependent sigmoid function. **(E)** Simulated and observed *SUC2* expression. The simulated data was obtained by simulating 10,000 cells from the feedback mediated model. **(F)** Simulated deletions obtained by simulating 10,000 cells for each case from the feedback mediated model. Simulated intensity was sampled at discrete time-points. Error bars represent the 0.20 and 0.80 quantiles within the simulated single cell population.

The feedback mediated model (Figure 4D) accounts for phosphorylated and dephosphorylated forms of nuclear Mig1 as well as for observed movement of Mig1 out of the nucleus upon a glucose downshift. The model consists of five states, phosphorylated (active) SNF1-complex (*SNF*1*p*), nuclear Mig1 (*Mig*1), phosphorylated nuclear Mig1 (*Mig*1*p*), *SUC2* and a feedback mediating component (*Y*). All rate-equations, excluding Mig1 nuclear transport, were constructed using mass action and Hill kinetics (Equation 3). A sigmoid function, σ(*SNF*1*p*), was implemented and parameterized to match observed Mig1 behavior (Equation 4b, for parametrization details see Figure S3), so that Mig1 stays in the nucleus during low SNF1 activity (Figure 4A), and that roughly as much Mig1 moves out of the nucleus when the external glucose is reduced from 4 to 1.5%, as when it is reduced from 4 to 0.1% (Bendrioua et al., 2014). The model investigates if a recovery in intracellular energy, resulting in a decrease in *SNF*1*p* activity (Mayer et al., 2011), can explain the observed *SUC2* expression. This is achieved by including the likely recovery in intracellular energy levels, which is an effect of genes activated upon glucose starvation and the increased SNF1p-activity (which results in increased *SUC2* expression) (Hedbacker and Carlson, 2008), via the production of *Y* (Equation 3e). Furthermore, as a partial recovery in energy levels should result in reduced SNF1-complex activity and hence *SUC2* activity, *Y* inhibits formation of *SNF*1*p* (Equation 3a).

(3a) $$\begin{array}{r} \frac{\text{d}SNF1p\left(t\right)}{\text{d}t}=k_{1}-k_{\text{ex\_glc}}\left(t\right)A\left(t\right)SNF1p\left(t\right)-k_{10}Y\left(t\right)SNF1p\left(t\right) \end{array}$$

(3b) $$\begin{array}{l} \frac{\text{d}Mig1(t)}{\text{d}t}=k_{2}-k_{3}SNF1p(t)Mig1(t)+k_{4}Mig1p(t)\\ \;-k_{5}(1+\sigma (SNF1p(t)))Mig1(t) \end{array}$$

(3c) $$\begin{array}{l} \frac{\text{d}Mig1p(t)}{\text{d}t}=k_{3}SNF1p(t)Mig1(t)-k_{4}Mig1p(t)\\ \;-k_{5}\left(1+\sigma \left(SNF1p(t)\right)\right)Mig1p(t) \end{array}$$

(3d) $$\frac{\text{d}SUC2(t)}{\text{d}t}=\frac{k_{6}}{K+Mig1(t-\tau_{\text{m}})}-k_{7}SUC2(t)$$

(3e) $$\frac{\text{d}Y(t)}{\text{d}t}=k_{8}\left(SUC2(t)-SUC2_{t_{0}}\right)-k_{9}Y(t)$$

(3f) $$\begin{array}{r} SNF1p\left(t\right)=0,\;t=t_{0} \end{array}$$

(3g) $$\begin{array}{r} Mig1\left(t\right)=Mig1_{t_{0}}\;\forall t\in \left[t_{0}-\tau_{\text{m}},t_{0}\right] \end{array}$$

(3h) $$\begin{array}{r} Mig1p\left(t\right)=0,\;t=t_{0} \end{array}$$

(3i) $$\begin{array}{r} SUC2\left(t\right)=SUC2_{t_{0}},\;t=t_{0} \end{array}$$

(3j) $$\begin{array}{r} Y\left(t\right)=0,\;t=t_{0} \end{array}$$

The initial values for phosphorylated *Mig*1*p* and *SNF*1 were set to 0 as the model is in rich glucose conditions at time zero. By the same arguments as for the simple feedback model, the initial value for the feedback component (*Y*) was fixed to 0, the YFP-maturation was represented by a time-delay, and a steady state which is broken by a reduction in the external glucose signal was enforced at time zero (Equation 4a). To ensure an initial steady state for *SNF*1*p*, it was noted that the external glucose signal should be heavily amplified (A) during high glucose conditions (Equation 4a and Table S2), as multiple processes negatively regulates *SNF*1*p* during high glucose conditions (Ruiz et al., 2011, 2013; Zhang et al., 2011; McCartney et al., 2016). Furthermore, by assuming a steady state expression for the initial values of *Mig*1 and *SUC*2 were obtained as a function of rate parameters (Equation 4c), and an expression of *k*_glc_ext_(*t*) was obtained as a function of *k*_1_ (Equation 4a). Lastly, as the simple model suggested a delayed feedback, this was added in the model by making the feedback dependent on the YFP activity of the Snf1/Mig1 regulated *SUC2* gene. In addition to the information presented here, a more detailed description of each state and accompanying rate equations is presented in Table S2.

(4a) $$\begin{array}{l} k_{\text{ex}\_\text{glc}}(t)A(t)SNF1p(t)\\ =\left\{ \begin{array}{l} kGlc^{\text{ext}}\underset{\approx 1}{\underset{︸}{A(t)SNF1p(t)}}\approx kGlc^{\text{ext}}\overset{\text{steady state}}{=}k_{1},\;t<0.0483\\ k\frac{Glc^{ext}}{40}\underset{=1}{\underset{︸}{A(t)}}SNF1p(t)=\frac{k_{1}}{40}SNF1p(t),\;t\geq 0.0483 \end{array}\right. \end{array}$$

(4b) $$\sigma \left(SNF1p(t)\right)=\frac{1}{1+\text{exp}\left(-3\left(SNF1p(t)-4.5/3\right)\right)}$$

(4c) $$\begin{array}{l} Mig1_{t0}=\frac{k_{2}}{k_{5}\left(1+\sigma \left(SNF1p(t_{0})\right)\right)}\\ SUC2_{t_{0}}=\;\frac{k_{6}}{(K+Mig1_{t0})k_{7}} \end{array}$$

When fitting the model to the *SUC2* expression data all unknown parameters, except one which was fixed (*K* = 0.1), were estimated with a full random effects covariance matrix Ω. This approach reduces the size of Ω and consequently ensures acceptable standard errors of the estimated parameters (Table S5). The unidentifiable Hill-coefficient (Equation 3d) was fixed to the smallest integer that produced a good fit (*n* = 1). In addition to the information presented here, inference details for each parameter is presented in Table S2.

It should be noted that the reaction-scheme in Figure 4D can also be modeled by an approach that requires less assumptions, and is more consistent with the feedback cascade model (Equation 1). This by replacing $k_{\text{ex}\text{\_}\text{glc}}ASNF1p$ with; −*k*_*ex*_glc_−*k*_11_*SNF*1*p*, a steady state argument will result in the same expression for *k*_*ex*_glc_ as in Equation (4a) (*k*_ex_glc_ = *k*_1_, *t* <0.0483 and *k*_ex_glc_ = *k*_1_/40, *t*≥0.0483). Overall, this approach also produces a good fit (Figure S4A), with the same dynamic characteristics as in the feedback mediated model, e.g., Mig1 partially relocates back into the nucleus upon long term glucose starvation (Figure S4B). However, this approach also results in a non-identifiable model, since by adding the *k*_11_ rate-parameter an additional 12 parameters have to be estimated due to the random effects covariance matrix. Hence, we used the model presented in Equation (3) as it is identifiable, is biologically justifiable (Table S2), captures key biological features, such as having practically zero *SNF*1*p* activity during high glucose (McCartney and Schmidt, 2001), and also keeps the dynamics of the approach with less assumptions.

Furthermore, it should be noted that the SNF1 related state variables in the two models correspond to different entities. In the feedback cascade model (Figure 3A), *SNF*1*pat* represents the inhibitory activity of the Snf1/Mig1 system. The inhibitory, instead of the activating activity, of the Snf1/Mig1 pathway was implemented in the model as the Snf1/Mig1 pathway generally is reported to inhibit the activity of the *SUC2* promoter (Broach, 2012). As the feedback mediated model (Figure 4D) is more detailed regarding the Snf1/Mig1 pathway, the SNF1 related state variable *SNF*1*p* represents the activity of phosphorylated SNF1-complex. The reason for using *SNF*1*p*, instead of *SNF*1 (non-phosphorylated complex), is 2-fold. Firstly, our data show how *SNF*1*p* affects Mig1 nuclear export (Figure 4A). Secondly, Mig1 is phosphorylated in a *SNF*1*p*-dependent manner (Shashkova et al., 2017). However, it is not known how SNF1 affects dephosphorylation of *Mig*1*p*, hence a term *Mig*1*p*·*SNF*1 would not be justifiable to include in the model. As the SNF1 related state variables correspond to different entities, they reflect the amount of intracellular energy levels, which SNF1 senses via the ATP/ADP ratio (section 3.2), differently. More precisely, high intracellular energy levels is represented by high *SNF*1*pat* and low *SNF*1*p*, with the conversely holding for low intracellular energy levels.

### 2.7. Parameter Estimation

In an ODE-model where the unknown model parameters are allowed to vary between cells, the dynamics of cells *i* = 1, …, *n* are given by

(5) $$\frac{\text{d}x_{i}(t_{j})}{\text{d}t}=g\left(x_{i},k_{i},u(t_{j}),t_{j}\right),\;x_{i}(t_{0})=x_{i0},$$

where **g** is the reaction kinetics governed by the value of the model states **x_*i*_**, kinetic parameters **k**_*i*_ and potential input functions **u**(*t*). In NLME and the standard two stage (STS) approach, the goal is to estimate the unknown model parameters **b**_*i*_ = (**k**_*i*_, **x**_*i*0_) and their underlying parameter distribution using observed data **y** = (**y**_1_, …, **y**_*n*_). In the course of this work, the models (Figures 3A, 4D), were related to the observed *SUC2* expression data (Figure 2B) via, as in a previous study with similar data (Almquist et al., 2015), an additive error model

(6) $$\begin{array}{r} y_{i}\left(t_{j}\right)=y_{ij}=\underset{\text{simulated SUC}2}{\underset{︸}{{ŷ}_{ij}\left({\text{b}}_{i}\right)}}+e_{ij},\;e_{ij}\sim N\left(0,s^{2}\right), \end{array}$$

where *s*^2^ is the variance of the measurement error.

#### 2.7.1. Non-linear Mixed Effect Approach

In the non-linear mixed effect (NLME) approach, the unknown parameters are split into a fixed effect **b**, and an individual random effect η_*i*_. Here, the individual parameters were assumed to be lognormally distributed; ${\text{b}}_{i}=\text{b}e^{\eta_{i}}$, $\eta_{i}\sim N\left(0,\Omega \right)$. Lognormal parameters were assumed instead of normally distributed parameters by two reasons. Firstly, lognormality ensures positive parameters. Secondly, rate constants are generally products of different factors (Atkins et al., 2013). Thus, a lognormal distribution is more suitable than a normal distribution (Limpert et al., 2001).

The fixed effects, covariance matrix of random effect and the noise parameter, ψ = (**b**, Ω, *s*^2^), are estimated simultaneously in NLME by maximizing a likelihood function

(7) $$\begin{array}{r} L\left(\psi |\text{y}\right)=\overset{n}{\underset{i=1}{\prod }}\int p\left({\text{y}}_{i}|\eta_{i},\text{b},s^{2}\right)p\left(\eta_{i}|\Omega \right)\text{d}\eta_{i}. \end{array}$$

The integral in Equation (7) is generally intractable, and is computationally expensive to approximate (Davidian and Giltinan, 2003). Consequently, other methods than the classical optimization methods must be used to maximize the likelihood. Here the SAEM-algorithm (Kuhn and Lavielle, 2005), via the Monolix software (Lixoft, 2019), was used. The SAEM-algorithm was chosen as it works well for NLME-models (Chan et al., 2011), and it's convergence has been rigorously proven (Delyon et al., 1999). Given estimated population parameters $\hat{\psi }=\left(\hat{\text{b}},\hat{\Omega },ŝ\right)$, new cells were simulated by first randomly drawing $\text{a}\sim N\left(0,\hat{\Omega }\right)$, and then calculating the parameters corresponding to a simulated cell by ${\text{b}}_{\text{simulated}}=\hat{\text{b}}e^{\text{a}}$.

To construct diagnostic plots based on individual predictions (IPRED-plots Figures S1A, S2A), the empirical Bayes estimates (EBS:s) were used; ${\text{b}}_{i}^{\left(\text{EBE}\right)}=\mathrm{argmax}p\left({\text{b}}_{i}|\hat{\psi },{\text{y}}_{i}\right)$ (Davidian and Giltinan, 2003). To construct diagnostic plots of the parameter distributions (Q-Q plots Figure S1B), random samples from the conditional distribution *p*(ψ_*i*_|**y**_*i*_) were used instead of the EBE:s, avoiding potential effects of η-shrinkage on model diagnostics (Lavielle and Ribba, 2016).

Identifiability was investigated by calculating the standard errors of the estimated parameters. The standard errors were obtained by inverting the observed Fisher Information Matrix (FIM), which was calculated via a stochastic approximation algorithm implemented in Monolix (Lixoft, 2019). The method of profile likelihood (Raue et al., 2009), is typically superior to asymptotic approaches, such as FIM for investigating model identifiability. However, the computational burden of optimizing and evaluating the likelihood (Equation 7), makes the profile likelihood method too computationally expensive to be used here.

#### 2.7.2. Standard Two Stage Approach

In the standard two stage (STS) approach, **b_*i*_** is first estimated for each cell. Given individual estimates ${\hat{\text{b}}}_{i}$, the parameter distribution is inferred. The parameters were assumed to follow a lognormal distribution (section 2.7.1).

The individual parameters were estimated by the maximum likelihood method (Llamosi et al., 2016). To avoid a constrained optimization problem, and to facilitate a more efficient optimization (Raue et al., 2013), the parameters were estimated on the log-scale. Given the error model (Equation 6), the log-scale parameters **lb**_*i*_ are obtained by maximizing the log-likelihood

(8) $$\begin{array}{l} (l{\hat{b}}_{i},{\hat{s}}_{i})=\underset{{}_{lb_{i},s_{i}}}{\arg\max}-\frac{n_{i}}{2}\text{ln}s_{i}^{2}-\frac{1}{2\sigma_{i}^{2}}\sum_{j=1}^{n_{i}}(y_{ij}-{\hat{y}}_{ij}(lb_{i}){)}^{2}\\ \;-\underset{=\text{constant}}{\underset{︸}{\frac{n_{i}}{2}\text{ln}2\pi }}. \end{array}$$

To solve the optimization problem (Equation 8), the BOBYQA algorithm (Powell, 2009) was used.

Given estimated individual parameters on the log-scale ${\hat{\text{l}\text{b}}}_{i}$, simulated cells were obtained by: (1) Noting that as the model parameters are assumed to be log-normal, the log-scale parameters **lb**_*i*_ become multivariate normal. (2) Via the method of maximum likelihood estimating the parameters of this multivariate normal distribution

(9) $$\begin{array}{r} \hat{\mu }=\frac{1}{n}\overset{n}{\underset{i=1}{\sum }}{\text{l}\text{b}}_{i},\;\hat{\Sigma }=\frac{1}{n}\overset{n}{\underset{i=1}{\sum }}\left({\text{l}\text{b}}_{i}-\hat{\mu }\right){\left({\text{l}\text{b}}_{i}-\hat{\mu }\right)}^{\text{T}}. \end{array}$$

(3) generating simulated parameter vectors on the log-scale, that is a simulated cells, by randomly drawing vectors from ${\text{l}\text{b}}_{\text{simulated}}\sim N\left(\hat{\mu },\hat{\Sigma }\right)$.

#### 2.7.3. Comparison of STS and NLME Approach

Two parameters, *k*_5_ and *k*_8_, in the feedback cascade model (Figure 3A and Equation 1) were assumed to not vary between cells. Thus, they were estimated without random effect using the NLME-framework. There is no obvious, single, way to include parameters without random effect in the STS-approach. Thus, two different approaches were used for the STS-estimation. (i) All parameters were allowed to vary between cells. (ii) All parameters were allowed to vary in a first parameter estimation run. Then in a second run *k*_5_ and *k*_8_ were fixed according to the mean-values obtained in the first run. Approach (ii) yielded better results, as approach (i) to a larger degree overestimated the variability when simulating the population behavior (Figure S1F), and is thus used for comparison.

#### 2.7.4. Implementation Details

In the implemented NLME approach, the population parameter ψ, EBE:s, samples from *p*(ψ_*i*_|**y**_*i*_) and the standard errors were calculated using Monolix (version 2019R2) (Lixoft, 2019). For the STS approach, the individual parameters (Equation 8) were estimated using the BOBYAQ algorithm in the NLopt (version 0.5.1) library in the Julia (version 1.3.1) programming language (Powell, 2009; Bezanson et al., 2017; Johnson, 2020). To ensure a fair comparison between NLME and STS, the same starting values were used for both approaches. For the cascade feedback model (Figure 3A) starting values were obtained by choosing the parameters values which produced the best model fit to the observed mean value using a multiple shooting approach. For the feedback mediated model (Figure 4D) this approach was not feasible due to stability issues. Consequently, starting values were obtained by taking the population parameters obtained when running the SAEM algorithm on manually adjusted starting values. To simulate new cells, the DifferentialEquations (version 6.11.0) library in Julia was used for solving the delay-differential equation system that makes out the models (Rackauckas and Nie, 2017). All the calculations were performed on a Dell Latitude with eight cores [Intel(R) Core(TM) i5-8365U CPU @ 1.60 GHz] running on Ubuntu 18.04.4.

The code used to produce all results in this paper can be found on GitHub (https://github.com/cvijoviclab/SUC2_long_term_regulation). Efforts have been made to make the result as reproducible as possible by basing the directory structure on two suggestions (Noble, 2009; Wilson et al., 2017). Given a Unix-based operative system, Monolix (version 2019R2) and Julia (version 1.3.1) the results should be reproducible by running the Run_all-script. More details about reproducing the results can be found on GitHub.

## 3. Results

### 3.1. Long-Term Observation of *SUC2* Promoter Expression Reveals Regulation of Promoter Activity After Initial Activation

For a pathway to react appropriately to a stimuli it first needs to be activated, and thereafter the activation needs to be regulated according to the strength of the stimuli. For the Snf1/Mig1 pathway, the current understanding can only result in the monotonic behavior of the activation (Welkenhuysen et al., 2017). That is, if a cell is presented with glucose depletion Mig1 leaves the nucleus and remains there until glucose is available again in the cellular environment. However, recent long-term continuous observation of Snf1/Mig1 pathway has shown pulsatile behavior over an extended period of glucose availability (Dalal et al., 2014; Lin et al., 2015). To elucidate the mechanism behind the long-term adjustment of the Snf1/Mig1 pathway activity upon glucose depletion yeast cells grown on 4% glucose were exposed to a shift to 0.1% in a microfluidic device. These cells contained a construct carrying YFP behind a *SUC2* promoter, allowing for measurement of the *SUC2* expression used to calibrate the models (Figure 2A). The *SUC2* gene encodes for two types of invertase, a secreted glycosylated form and an intracellular, non-glycosylated form. The former is regulated by the Snf1-pathway while the latter is produced constitutively in small amounts compared to the glycosylated form (Carlson and Botstein, 1982). Therefore, the non-glycosylated form was not considered in the developed models. The fluorescence intensity was observed for 480 min with an interval of 5 min, yielding a data-set with a rich amount of data points and cells (124 cells with 97 data points each). Initially the level of fluorescent intensity increased 212% on average (Figure 2B), thereafter between time points 180 and 210 min the signal started to decline. The decline of signal occurs when the production of new protein is smaller then the turn-over of the YFP protein through breakdown and bleaching. Hence, the decline implies that a decrease of *SUC2* promoter activity has taken place. This suggests that after an initial activation time a negative feedback takes place in the nutrient signaling network which reduces the expression of the *SUC2* promoter.

### 3.2. Modeling Suggests Delayed Negative Feedback Due to a Partial Recovery in Intracellular Energy Levels After Initial Activation

The single-cell *SUC2* expression data suggests the existence of a negative feedback, which upon long-term glucose starvation reduces the *SUC2* expression (Figure 2B). A possible source of this feedback is a partial recovery in the intracellular energy levels, that is an increase in cellular metabolism which results in an increase of the ATP/ADP ratio which further regulates the SNF1 complex. More specifically, during low intracellular energy levels the SNF1-complex is bound by ADP and protected (sterically) against deposphorylation, while the phosphorylation of the complex occurs continuously (Rubenstein et al., 2008; Chandrashekarappa et al., 2013). Conversely, during rich intracellular energy levels, e.g., rich glucose conditions, SNF1 is not ADP bound and is exposed to dephophorylation resulting in low SNF1 activity (Mayer et al., 2011). Hence, a partial recovery in intracellular energy levels could explain the SUC2-expression, as it should be reflected by an increased inhibitory activity of the Snf1/Mig1 pathway (decreased inhibitory activity on Mig1 by the SNF1 complex), ultimately resulting in reduced *SUC2*-expression. Furthermore, a partial recovery in intracellular energy levels is expected upon long-term glucose starvation. For example, upon starvation reduced Snf1/Mig1 pathway inhibitory activity results in metabolism of alternative carbon sources and turns off energy producing processes (Hedbacker and Carlson, 2008).

To investigate if feedback mechanism via energy levels is mechanistically possible, we constructed a simple dynamic model (Figure 3A and Equation 1). The potential recovery in energy levels, which is a consequence of genes activated by glucose starvation and low Snf1/Mig1 pathway inhibitory activity, was modeled via the production of the feedback cascade. Furthermore, as a recovery in energy levels should result in increased Snf1/Mig1 pathway inhibitory activity, the feedback cascade was modeled to promote inhibitory activity. When fitted using a NLME approach, the model captures the single-cell behavior of the *SUC2* expression. More specifically, the model captures the observed individuality (Figure S1A) and also, by sampling from the estimated parameter distribution, is capable of simulating the observed population behavior (Figure 3B). An interesting model parameter is the feedback time-delay, τ_f_, whose estimated distribution is separated from zero (Figure S1C). This suggests a delayed feedback with respect to the glucose downshift.

Overall, the feedback cascade model (Figure 3A) is able to explain the observed *SUC2* reduction upon long-term glucose starvation. As the feedback is modeled to behave as a partial recovery in intracellular energy levels, the feedback might act mainly via Snf1. However, due to the simplicity of the model it is not possible to deduce if this is a sufficient mechanism, e.g., the feedback might also act via Mig1 (Figure 1).

#### 3.2.1. NLME Outperforms STS for Data Rich in Observations

Considering that parameter estimation for single-cell time-lapse data is challenging, we explored and compared the performance of standard two stage (STS) (Karlsson et al., 2015) and non-linear mixed-effect (NLME) (Almquist et al., 2015; Karlsson et al., 2015; Llamosi et al., 2016; Fröhlich et al., 2019; Marguet et al., 2019) approaches. NLME is considered superior to STS when the data is not rich (Karlsson et al., 2015). However, the experimental *SUC2* data obtained in this work can be viewed as sufficiently rich according to the criteria used by Karlsson et al. (2015). This is because the noise appears small, as there is a clear signal to noise ratio (Figure 2B) and furthermore, the data is not sparse (124 cells, with 97 non-zero observations each). Consequently, first fitting the simple feedback model to each cell, and from the fitted parameters infer the population parameters might yield as accurate parameter estimates, as the more advanced NLME-framework (Karlsson et al., 2015). It should be noted that when a system is perturbed by an external stimuli that yields a small effect, NLME outperforms the STS-approach (Karlsson et al., 2015). However, the observed *SUC2* data has no such stimuli, making this criteria irrelevant here.

Although both approaches appear to produce almost equally good individual fits (NLME slightly is better) to the observed *SUC2* data (Figures S1A,D), our analysis suggests that the STS-approach to a larger degree overestimates the variability when simulating the observed population behavior (Figures 3B,C). Furthermore, the STS fit does not capture the decrease in intensity for upper quantile equally well as the NLME approach. This suggests that the STS-approach estimates the parameter distribution incorrectly. Consequently, the STS-estimated distribution is unsuitable to use for further analysis, like model extrapolations. This is non-ideal from a computational perspective, as the computational times vary for two approaches (2.2 h to run the easily parallelizable STS estimation on a single core, compared to 5.0 h for the NLME estimation on eight cores). The difference when simulating the population behavior, might be due to outliers in the individual parameter estimates for the STS-approach. For example, the distribution assumption of *k*_6_ is violated due to outliers in the STS-approach (Figure S1E).

Overall, the STS-approach is less suitable compared to NLME when simulating the observed population behavior, despite that both approaches have almost equally good individual fits.

### 3.3. SNF1 Is Central in Regulating *SUC2* Expression and Mig1 Nuclear Localization

The feedback cascade model (Figure 3A), does not reveal on which components of the Snf1/Mig1 pathway a *SUC2-*regulating feedback might act, but suggests a partial recovery in intracellular energy levels. Thus, a candidate is that the feedback acts mainly via the believed to be energy regulated (Rubenstein et al., 2008; Chandrashekarappa et al., 2013), SNF1-complex. A criteria for the feedback to act via SNF1, is that *SUC2* should be strongly regulated in a SNF1-dependent manner. To investigate this, we examined *SUC2* expression by measuring invertase activity under glucose rich and limited conditions (Figure 4B). Wild type (WT) cells showed an increased amount of glucose formed under glucose depletion compared to high glucose conditions. Absence of the *SNF1* gene resulted in decrease of the invertase activity regardless of the glucose presence. This supports previous findings that *SUC2* expression is regulated in a SNF1-dependent manner (Carlson et al., 1981; Neigeborn and Carlson, 1984). To be able to construct a model that examines the possibility of a *SUC2* regulating feedback acting via the SNF1-complex, we investigated the role of Snf1 in Mig1 localization. This was done by examining Mig1 localization when Snf1 kinase activity is inhibited (Figure 4A). We introduced *SNF1-I132G*, a PP1 analog-sensitive version of Snf1, into the cells with genomically integrated Mig1-GFP fusion as well as Nrd1-mCherry as a nuclear localization reporter. An *I132G*mutation at the ATP-binding pocket of Snf1 generates a novel structure sensitive to 1NM-PP1, an ATP competitive kinase inhibitor (Knight and Shokat, 2007; Rubenstein et al., 2008). Functionality of the analog-sensitive version of Snf1 has already been previously reported (Rubenstein et al., 2008; Shashkova et al., 2017). Upon glucose limitation, incubation of cells expressing *SNF1-I132G* with 25 μM 1NM-PP1 resulted in Mig1 retention in the nucleus, while the wild type Snf1 was irresponsive to the inhibitor. This is consistent with previous observations that Snf1 kinase activity is key for Mig1 nuclear export (Shashkova et al., 2017; Wollman et al., 2017).

Overall, experimental data suggests that *SUC2* is regulated in a SNF1-dependent manner. Furthermore, our data confirms that Snf1 activity is key for Mig1 nuclear export (Shashkova et al., 2017).

### 3.4. Modeling Reveals Potential Feedback Mechanism via Phosphorylated SNF1 After Initial Activation

Having the Mig1 localization and invertase activity data (Figures 4A,B), we were able to investigate the hypothesis that *SUC2* is mainly regulated in a SNF1-dependent manner upon long term glucose starvation by constructing a new dynamic model (Figure 4D and Equation 3). Considering that *SUC2* is regulated by SNF1 via Mig1 (Figure 1) we included the phosphorylated and dephosphorylated forms of nuclear Mig1. Further, it has been observed that upon a glucose downshift, a majority of Mig1 moves out of the nucleus (Treitel et al., 1998; Delyon et al., 1999), however, the mechanism behind this behavior is not known. To account for this observation, the transport behavior of Mig1 was included into the model by a sigmoid function (Equations 3b,c), which was parameterized to match observed Mig1 behavior (Figure S3). The potential partial recovery in energy levels, which is a consequence of genes activated by glucose starvation and high phosphorylated SNF1 activity (Hedbacker and Carlson, 2008), was modeled via the feedback mediating component whose production is promoted by high expression of Snf1/Mig1-controlled genes. Lastly, owing to available data (Figure 4), knowledge of the Snf1/Mig1 system (Shashkova et al., 2017), and that this model is more detailed regarding the Snf1/Mig1 pathway compared to the feedback cascade model (Figure 3A), the SNF1 related state variable was modeled to correspond to phosphorylated SNF1 (for detailed motivation see section 2.6). Thus, intracellular energy levels are reflected in the model by the activity of phosphorylated SNF1 (section 3.2). Hence, the feedback component which is promoted by a partial recovery in intracellular energy levels is modeled to inhibit the SNF1 related state variable (Figure 4D), in contrast to feedback cascade model where the feedback promotes inhibitory Snf1/Mig1 activity (Figure 3A).

Similarly to the first model, this model captures the observed individuality in *SUC2* data using a NLME approach as well as observed population behavior (Figure 4E and Figure S2A). It further captures that total nuclear Mig1 (phosohorylated + dephosphorylated) moves out of the nucleus upon glucose starvation (Figure S2B). Also, the model suggests that the total amount of nuclear Mig1 partially recovers upon long term glucose starvation (Figure S2B), which has been shown before (Dalal et al., 2014; Lin et al., 2015), due to decrease in nuclear export rate owing to the feedback acting on phosphorylated SNF1 (Figure S2C). Deleting the SNF1 component in the model by setting it to zero, leads to a constant low *SUC2* expression upon glucose starvation (Figure 4F). This matches the experimentally observed low *SUC2* activity when SNF1 is deleted (Figure 4B). However, the model suggests higher *SUC2* expression than observed in experimental data under the same conditions. To see further effects of deletions in the model, the ability of the model to mediate the feedback was removed by setting the feedback mediating component *Y* to zero (Figure 4F). This resulted in a strong increase of *SUC2-*expression compared to wild type. Deleting SNF1 and feedback mediating component yields the same result as when only deleting SNF1. This is expected, as the model assumes external glucose signals to be mediated to *SUC2* solely via the SNF1 complex.

Overall, the feedback mediated model (Figure 4D) is able to explain the experimental data reporting a reduction in *SUC2* expression upon long-term glucose starvation. As phosphorylated SNF1 is regulated via intracellular energy levels (section 3.2), this suggests that *SUC2* expression decreases due to a partial recovery in intracellular energy levels.

## 4. Discussion

Nutrient sensing pathways are playing an important role in cellular response to different energy levels. Current understanding of this response only results in monotonic behavior of the *SUC2-*promoter upon starvation. However, our single-cell microfluidics data show that *SUC2* expression decreases in the long-term. To investigate the regulation of the *SUC2-*promoter upon long-term glucose starvation we have combined fluorescence microscopy and microfluidics data together with non-linear mixed effect modeling. The single-cell time-lapse data show that after an initial activation time a negative feedback takes place in the nutrient signaling network reducing the expression of the *SUC2* promoter (Figure 2B). Our invertase assay and microscopy data confirm that *SUC2* is regulated in a SNF1-dependent manner and that Snf1 activity is key for Mig1 nuclear export (Figures 4A,B). Finally, we propose via dynamic modeling that the decrease in SUC2 expression is due to a partial recovery in intracellular levels which results in a feedback that acts on the SNF1-complex (Figures 4D,E).

Without feedback, the *SUC2* would continue to increase, resulting in an excessive amount of invertase protein considering the energy supply and demand of the cell. This would result in the uneconomic use of cellular resources. Therefore, it is necessary to regulate the level of *SUC2* expression according to the cellular demand. Signals arising from changes in metabolic flux can be used to regulate invertase production (Litsios et al., 2018). Our feedback mediated model suggests that the expression of the target genes in the Snf1/Mig1 pathway is regulated through a feedback loop acting via a potential feedback mediating component (*Y*) (Figure 4D). We suggest that this component controls energy supply, produced through cellular metabolic flux. By coupling the metabolic flux with the production of enzymes, a stable and fast re-balancing of the enzymatic protein supply and demand can be created, which leads to optimal energy homeostasis. As it has been shown that the ratio of ADP/ATP in the cell controls the dephosphorylation of Snf1 in yeast (Mayer et al., 2011; Xiao et al., 2011; Chandrashekarappa et al., 2013), this ratio could be the sensor which couples metabolic flux (energy levels) in the cell with production of invertase, and thereby the mechanism responsible for the negative feedback. Also, other known mechanisms could be responsible for the negative feedback, such as the Reg1 phosphorylation by PKA, which activity is controlled by secondary messenger and ATP-derivative cAMP (Castermans et al., 2012). Further could the negative feedback also involve other proteins targeted by the Snf1 pathway, such as Cat8, Adr1, and Sip4. These proteins are directly phosphorylated by Snf1 kinase and the expression of the encoding genes is controlled by Mig1 (DeVit et al., 1997). Cat8, Adr1, and Sip4 are involved in the cellular reprogramming during the diauxic shift and through this role influence the cell energy-metabolism (Vincent and Carlson, 1998; Haurie et al., 2001; Young et al., 2003). The alteration caused in the metabolism by these protein could be pivotal in the changing behavior of the *SUC2* expression.

The Glc7-Reg1 phosphatase is required for Snf1 dephosphorylation (Rubenstein et al., 2008). At the same time, Snf1 itself acts on Reg1 and prevents its association with the Glc7 subunit for the formation of the functional phosphatase (Sanz et al., 2000). This loop makes Reg1 a potential candidate to be involved in the energy regulated feedback proposed by our modeling (Figure 4D). Our simulations on cells deficient in potential feedback mediated component (*Y*) suggest an increase in the *SUC2* expression compared to the wild type (Figure 4F). We tested how the target genes are affected in yeast cells carrying the *Reg1* deletion. Our experimental data suggests an increase in invertase activity upon *REG1* deletion compared to the WT (Figure 4C). Furthermore, invertase activity on the cells without both Reg1 and Snf1 shows reduced invertase-activity, which is in agreement with the reduced *SUC2* expression when deleting *SNF1* and feedback mediated component (*Y*) in the model. This suggests that Reg1 is a central part of the feedback. However, as the feedback mediation in the model encompasses all potential energy regulated components that affects SNF1 activity, more components than Reg1 are likely involved in the feedback.

Our feedback meditating model suggests that the fine-tuning of expression, after the initial strong activation, of the target genes in the Snf1/Mig1 pathway is regulated through a feedback loop acting via a potential feedback mediating component (*Y*). Mig1 regulates genes essential for utilization of carbon sources (Lutfiyya et al., 1998), hence, Mig1 participates in controlling energy metabolism in the cell. This is another evidence supporting Reg1 being central part of the feedback mediated component as the intracellular energy levels have been shown to play an important role in the activation of Snf1, thus, its communication with the Glc7-Reg1 phosphatase (Rubenstein et al., 2008).

Deletion of Snf1 in the feedback mediated model resulted in a stable *SUC2* expression at a level similar to simulated wild type at time 0, which corresponds closely to *SUC2* expression at 4% glucose (Figure 4F). However, the invertase activity assay shows that the activity in the *SNF1* deletion strain is lower then the WT at 4% glucose (Figure 4B). This highlights that the model does not fully capture the behavior of the *SUC2* expression at 4% glucose. A probable cause of this discrepancy is the simplicity of the model. Other pathways have been shown to influence the *SUC2* expression and cross-talk has been suggested to be ubiquitous in the nutrient signaling system (Kayikci and Nielsen, 2015; Shashkova et al., 2017). A larger model, opposed to the small-scale model in this work, could include other pathways known to be able to influence the expression of *SUC2*. Larger mathematical models have been made (Kayikci and Nielsen, 2015; Welkenhuysen et al., 2018), however they are Boolean models and consequently cannot capture the dynamic, single-cell behavior of cells exposed to several environmental conditions. Due to this inherit drawback of Boolean models and the connectivity of the nutrient sensing pathways, a large-scale single-cell model is probably needed to fully understand the Snf1/Mig1 pathway dynamics.

Parameter estimation would pose a considerable challenge in constructing a large scale, mechanistic single-cell model of the nutrient sensing network. Here, we compared two estimation methods for single-cell time-lapse data, STS and NLME. Although our data is rich in observations, NLME outperformed STS when estimating the population parameter distribution (Figure 3). As discussed by Almquist et al. (2015), this is probably due to some cells not carrying sufficient information to properly estimate all parameters. Hence, parameters like *k*_6_ can take extreme values for certain cells (Figure S1E), ultimately resulting in bad estimates of the population parameters. Our result thus highlight that data rich in observations, is not equal to data that is optimally sampled for each cell. Consequently, it is far from guaranteed that STS, although it happens (Karlsson et al., 2015), equals NLME in performance for observation rich data. This fact, combined with previous underperformance (Almquist et al., 2015; Llamosi et al., 2016), suggests that the STS-approach is not a preferred method. However, this does not mean that the current NLME framework should be the preferred method for a large-scale model built on time-lapse data. For example, here we show that NMLE is computationally demanding even for a small model. The global two-stage (GTS) approach has been proposed as an alternative to NLME (Dharmarajan et al., 2019). However, GTS currently cannot handle multi-experiment data (Loos and Hasenauer, 2019), and it is questionable if a large model can be calibrated using single-experiment data. Overall, these shortcomings highlight that further development in parameter estimation methods is required for constructing large-scale mechanistic single-cell models.

In summary, our systems biology approach suggests that *SUC2* expression decrease upon long-term glucose starvation is due to a partial recovery in intracellular energy levels acting on the SNF1-complex.

## Data Availability Statement

The datesets presented in this study can be found in FigShare repository. The *SUC2* expression data for this study: https://figshare.com/s/d846d38177821c3a2c4e. The time-lapse microscopy images for this study: https://figshare.com/s/6544469f4cf99fc0d862.

## Author Contributions

SP developed the mathematical models, performed the simulations, and mathematical analysis. NW and SS planned and performed the experimental part of the work. NW and MC conceived the research. SP, NW, SS, and MC wrote the paper. All authors contributed to the article and approved the submitted version.

## Conflict of Interest

The authors declare that the research was conducted in the absence of any commercial or financial relationships that could be construed as a potential conflict of interest.
