# Supplementary material for: Fine-Tuning of Energy Levels Regulates SUC2 via a SNF1-Dependent Feedback Loop
Source: Front Physiol. 2020 Aug 14;11:954. doi: 10.3389/fphys.2020.00954 (PMC7456839; doi:10.3389/fphys.2020.00954)
Supplement: Supplementary file 1 [file Data_Sheet_1.pdf]

Supplementary Material

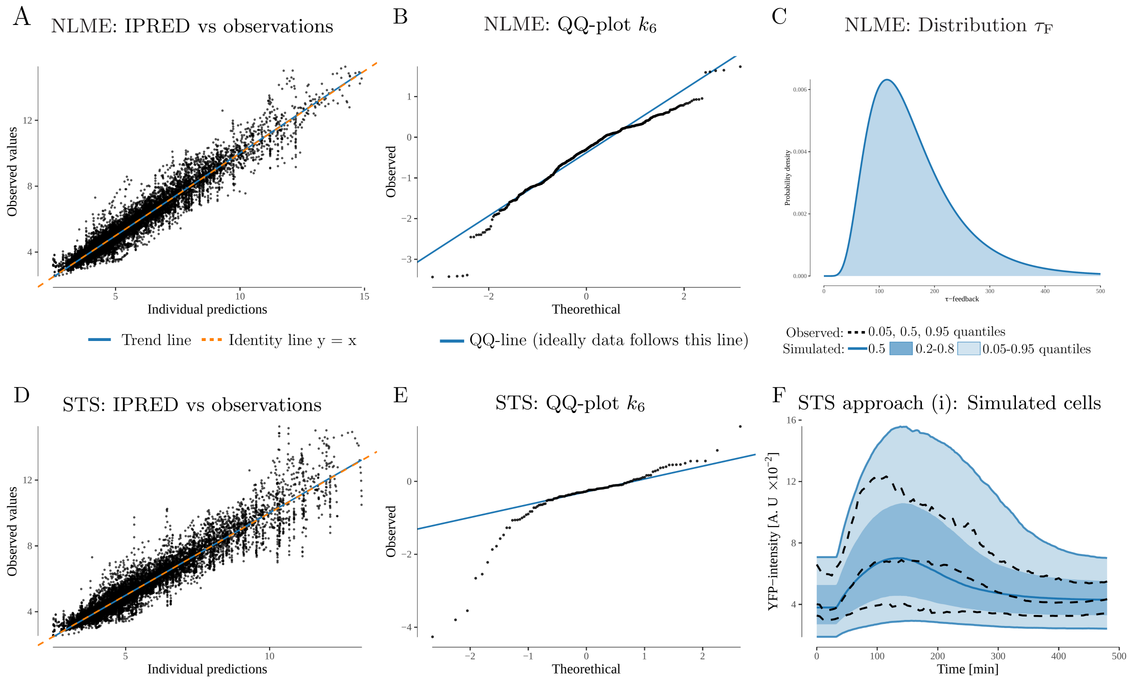

Figure S1. STS versus NLME for the feedback cascade model. (**A**, **D**) IPRED-plot, individual observations vs observed individual values. Ideally, the data should fall symmetrically around the identity line $y = x$. The IPRED plot for the NLME case should not be misgiving as ϵ-shrinkage = 0.066 (Karlsson and Savic, 2007) (**B**, **E**) Normal QQ-plot of $\ln(k_6)$. (**C**) Estimated distribution of τ_F for the NLME-fit. Noteworthily, the distribution is practically separated from zero. (**F**) Simulated and observed population behavior of *SUC2*-intensity when using STS approach (i); all parameters were allowed to vary between cells. The simulated data was generated by simulating $10\,000$ cells using the estimated parameter distribution for STS approach (i).

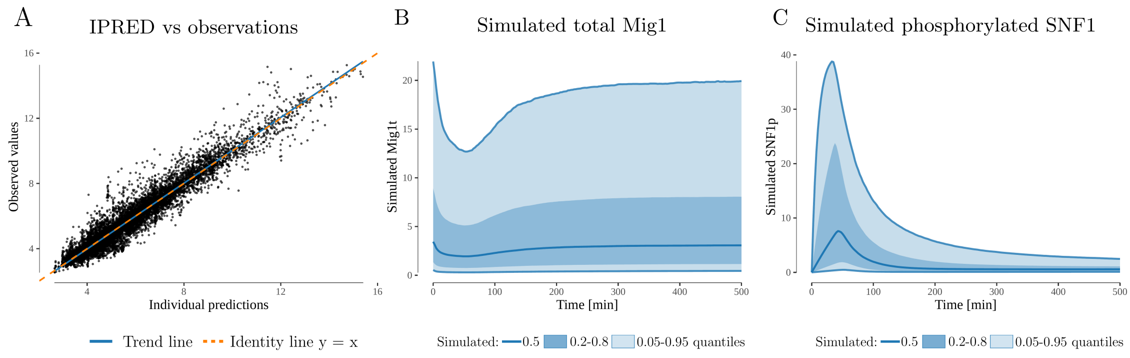

Figure S2. The feedback mediated model. **(A)** IPRED-plot, individual observations vs observed individual values. Ideally, the data should fall symmetrically around the identity line $y = x$. The plot should not be misgiving as ϵ-shrinkage = 0.039 (Karlsson and Savic, 2007) **(B, C)** Simulated total nuclear Mig1 (phoshorylated + dephosphorylated) and SNF1p. The simulated data was obtained by simulating $10\,000$ from the NLME-estimated parameters of the feedback mediated model.For (**B**) the long term increase in total nuclear Mig1 is due the feedback decreasing the $SNF1p$ activity (A). More precisely, owing to the feedback $SNF1p$ decreases below 2.667 (Fig. S3A). Thus the Mig1 nuclear export rate, which is modeled via the output of the sigmoid function (Eq. 3), decreases resulting in a net increase in total nuclear Mig1.

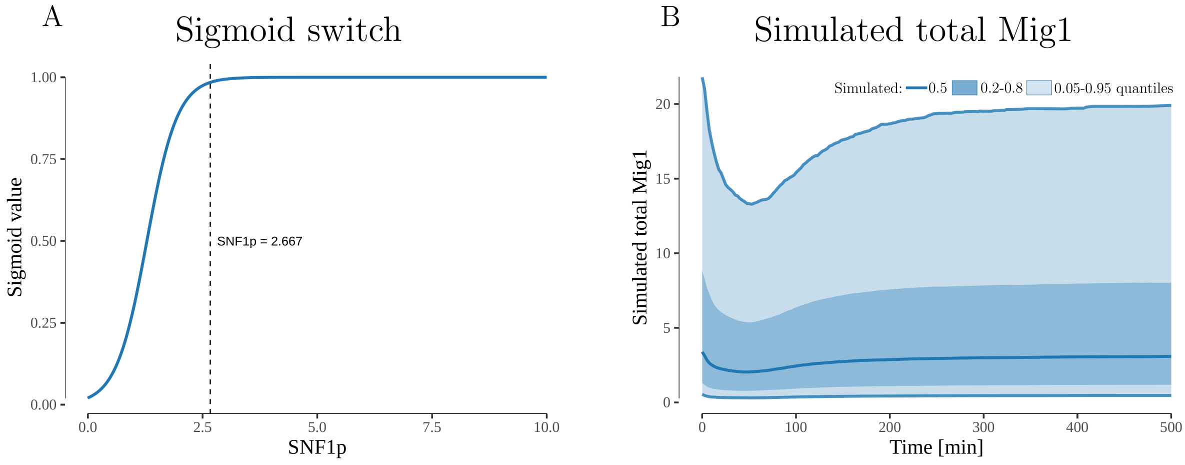

Figure S3. Properties of the sigmoid switch, $\sigma(SNF1p)$, used to model the export of Mig1 out of the nucleus. (**A**) The sigmoid function was parameterized so that Mig1 should stay in the nucleus during low $SNF1p \approx 0$ activity (Fig. 4A), and move out when the external glucose is reduced from $4 \rightarrow 1.5$ (cut by a factor of 2.667) (Bendrioua et al., 2014). To achieve this, the sigmoid function was calibrated to result in a full net transport of Mig1 out of the nucleus when the SNF1p value exceeds β, where $\beta = \max(SNF1p)$ under conditions where the glucose is cut by $\alpha = 2.667$. As Y response is delayed, $\max(SNF1p) = \alpha$ (set the derivative to zero after the glucose cut in the first model equation). Hence the sigmoid function was calibrated so that Mig1 moves almost fully out of the nuclues when $SNF1p > 2.667$, by choosing the arguments $\sigma(SNF1p) = 1/\big(1 + \exp(-3(SNF1p - 4.5/3))\big)$. (**B**) Simulated total Mig1t (total nuclear Mig1) when the external glucose is cut by $\alpha = 2.667$. That Mig1 shuffles out highlights that the switch was calibrated correctly. It has been observed that in these conditions that Mig1 should recover fully and faster (Bendrioua et al., 2014). The model does not capture this as it only accounts for the long-term feedback triggered upon heavy glucose starvation (glucose $4\% \rightarrow 0.2\%$).

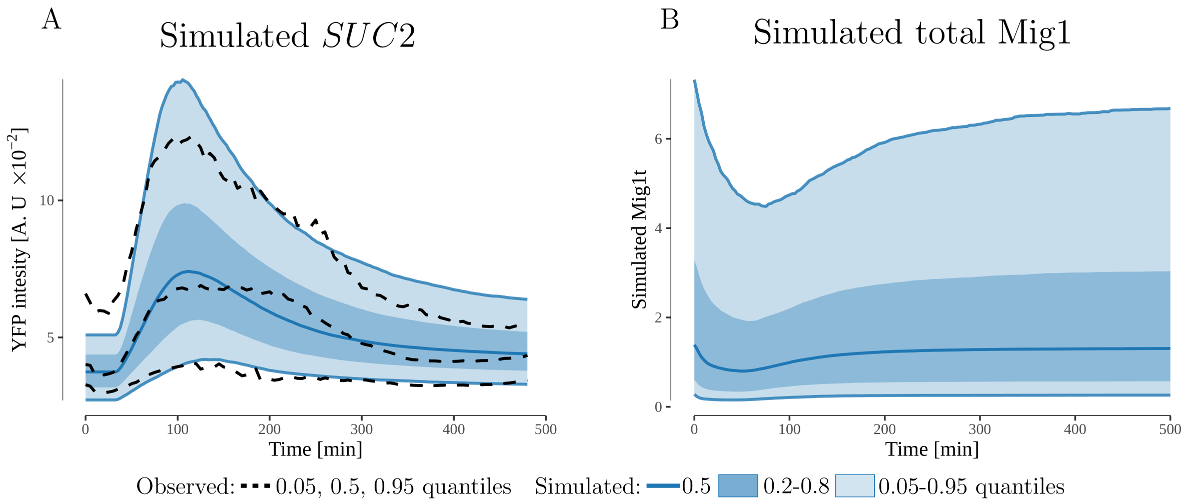

Figure S4. Alternative approach (section 2.6) for modeling the reaction scheme in Fig. 4D. (A) Simulated and observed SUC2. (B) Simulated observed total nuclear Mig1 (phoshorylated + non-phosphorylated). The simulations were generated by randomly drawing 10 000 cells from the NLME estimated population parameter distribution. For (B) the absolute values on the y-axis differ compared to Fig. S2B. However this is of small relevance, as in this study the qualitatively behavior of the Snf1/Mig1 pathway upon glucose starvation is of main interest.

Table S1. Description of each term and state in the feedback cascade model (Fig. 3A). In all terms, the reaction was assumed to be dependent on the specie(s) in the model that to our knowledge are known to be involved in the reaction.

**Snf1/Mig1 pathway inhibitory activity (SNF1pat)**		
State describing the inhibitory activity of the Snf1/Mig1 pathway (Fig. 1) on the *SUC2* promoter. The production of this state is promoted by glucose availability, as the Snf1/Mig1 pathway has a high inhibitory activity during glucose high intracellular energy levels, e.g rich glucose conditions (Hedbacker and Carlson, 2008). Note that this state only describes the inputs and outputs of the inhibitory activity of the Snf1/Mig1 pathway (Fig. 1).		
**Terms SNF1pat**	**Biological meaning and motivation**	**Notes on parameters**
$+k_{\text{ex\_glc}(t)}$	$k_{\text{ex\_glc}}(t)$: Rate governing the activation of SNF1pat via external glucose. The term actually corresponds to $kGlc^{\text{ext}}$. However, as $Glc^{ext} = \text{constant}$, it follows that $kGlc^{\text{ext}} = k_1$ when $t < 0.0483$ and $kGlc^{ext}/40 = k_1/40$ when $t > 0.0483$ (after external glucose cut).	k_1 estimated from data. Varies between individuals.
$-k_2SNF1pat(t)$	k_2: Rate governing the degradation of SNF1pat activity.	k_2 estimated form data. Varies between individuals.
$+k_3X(t-\tau_{\text{f}})$	k_3: Rate governing the production of SNF1pat activity via a feedback cascade. As X production is promoted by glucose starvation, this term mediates the energy regulated feedback on SNF1pat (increases SNF1pat activity upon long-term starvation) To include that the potential feedback acting on SNF1pat likely is a cascade, a time-delay τ_{f} was used (Fig. 3A). It is unknown which processes X encompasses, therefore first order kinetics were used to model the process.	k_3 estimated from data. Varies between individuals. τ_{f} estimated from data. Varies between individuals.
**Activity of the *SUC2* promoter**		
State describing the activity of the *SUC2* promoter. More precisely, the state corresponds to the measured YFP output of the *SUC2* promoter.		
**Terms *SUC2***	**Biological meaning and motivation**	**Notes on parameters**
$+\frac{k_4}{k_5+SNF1pat(t-\tau_{\text{m}})}$	k_4, k_5: Rates governing the inhibition of *SUC2*-promoter activity via SNF1pat activity. The mechanism that regulates the *SUC2* promoter is not known, hence the unidentifiable Hill-coefficient was set to the smallest value capturing the data n = 1. To ensure that the state corresponds to the measured YFP, the YFP maturation time was represented by a time delay τ_{m} (Fig. 3A)	k_4 estimated from data. Varies between individuals. k_5 Estimated from data. Constant between individuals τ_{m} fixed to 32 min Motivation in section 2.5.
$-k_6SUC2(t)$	k_6: Rate governing the degradation of *SUC2*-promoter activity (by turnover and bleaching).	k_6 Estimated from data. Varies between individuals.
**Potential feedback cascade (X)**		
State describing a potential feedback cascade activated by an external glucose drop. X (the feedback) is modeled to not have any influence during high glucose, and become activated upon a glucose shift. This is because the feedback is modeled to be dependent on the multiple genes that are activated upon starvation (low intracellular energy levels) (Hedbacker and Carlson, 2008). During glucose starvation, when activated, X is modeled to be inhibited by SNF1pat activity. This is because high SNF1pat activity corresponds to an increase in intracellular energy levels, which should reduce the need of a strong feedback signal. Since a multitude of energy saving genes, e.g genes responsible for metabolism of alternative carbon sources and for turning off fatty acid biosynthesis (Hedbacker and Carlson, 2008), are activated upon starvation, and SNF1pat is regulated via several energy-regulated processes, X corresponds to a lumping of several cellular processes.		
**Table continuous on next page**		

Terms X	Biological meaning and motivation	Notes on parameters
$+\mathcal{H}(t)\frac{k_7}{k_8+SNF1pat(t)}$	k_7, k_8: Rates governing the inhibition of X via SNFpat activity. As discussed above, X is activated by a glucose drop, this is modeled by $\mathcal{H}(t)$. As the mechanism that regulates X is not entirely known, the unidentifiable Hill-coefficient was thus set to the smallest value capturing the data.	k_7 estimated from data. Varies between individuals. k_8 Estimated from data. Constant between individuals.
$-k_9X(t)$	k_9: Rate governing the degradation of X activity.	k_9 estimated from data. Varies between individuals.

Table S2. Description of each term and state in the feedback mediated model (Fig. 4D). In all terms, the reaction was assumed to be dependent on the specie(s) in the model that to our knowledge are known to be involved in the reaction.

**Phosphorylated SNF1 (SNF1p)**		
State describing the activity/amount of the phosphorylated form of the SNF1 complex. As the phosphorylated form of SNF1 is mainly present during low intracellular energy levels, e.g low glucose conditions (McCartney and Schmidt, 2001), the initial value was set to zero.		
**Terms SNF1p**	**Biological meaning and motivation**	**Notes on parameters**
$+k_1$	k_1: Rate governing the production of SNF1p via the upstream kinases (UK). As the UK:s appear to have constant activity during high and low glucose availability (Rubenstein et al., 2008), the production was modeled as constant.	k_1 Estimated from data. Varies between individuals.
$-\underbrace{k_{\text{ex\_glc}}(t)}_{kGlc^{\text{ext}}}\mathcal{A}(t)SNF1p(t)$	$k_{\text{ex\_glc}}(t)$: Rate governing the breakdown of SNF1p via the external glucose signal. The breakdown is modeled to dependent on SNF1p, as during low glucose conditions growth of SNF1p should not be indefinite given deletion of Y, that is deletion of components that are likely central for the feedback such as Reg1 (Fig. 4B). Also, as there are a multitude of processes negatively regulating SNF1p during high glucose (McCartney et al., 2016) (Zhang et al., 2011; Ruiz et al., 2011, 2013), the initial steady state is modeled to be maintained by a glucose amplification factor; $\underbrace{SNF1p(t)}_{\ll 1}\underbrace{\mathcal{A}(y)}_{\gg 1} \approx 1$. Consequently, during high glucose the term reduces to $-kGlc^{\text{ext}}$. Given a steady state, this results in the strong but realistic model assumption, as $SNF1p \approx 0$ has been experimentally observed during high glucose (McCartney and Schmidt, 2001), that the external glucose signal is strong enough to maintain a very low (practically zero) SNF1p activity: $kGlc^{\text{ext}} = k_1$. The amplification term is modeled to disappear when the external glucose is reduced, as glucose/energy availability is a requirement for a multitude of regulating processes (Broach, 2012). Thus $\mathcal{A}(t) = 1$ when $t > 0.0483$. The factor Glc^{ext} is modeled to not disappear, as there should always be a basal level (likely governed by glucose availability) that negatively regulates SNF1p. Furthermore, during the external glucose drop the glucose is reduced by a factor 40, resulting in; $-kGlc^{\text{ext}}/40SNF1p(t) = k_1/40SNF1p(t)$. Overall, this yields the expression in Eq. 4.	$k_{\text{ex\_glc}}(t)$ fixed to k_1 by a steady state argument prior to glucose drop. Fixed to $k_1/40$ after glucose drop.
$-k_{10}SNF1p(t)Y(t)$	k_{10}: Rate governing breakdown of SNF1p activity via the feedback mediated component Y. As Y production is promoted by glucose starvation (see below), this term mediates the energy regulated feedback on SNF1p (decreases SNF1p activity upon long-term starvation). It is unknown which processes Y encompasses, therefore the process was modeled by first order kinetics	k_{10} Estimated from data. Varies between individuals
**Non-phosphorylated nuclear Mig1 (Mig1)**		
State describing the activity/amount of the non-phosphorylated form of nuclear Mig1.		
**Terms Mig1**	**Biological meaning and motivation**	**Notes on parameters**
k_2	k_2: Rate governing influx of Mig1 into the nucleus.	k_2 Estimated from data. Varies between cells
**Table continuous on next page**		

$-k_3 SNF1p(t)Mig1(t)$	k_3: Rate governing the influx of phosphorylated Mig1 into the nucleus. As Mig1 shuffles in and out of the nucleus regardless of glucose availability (Bendrioua et al., 2014), the influx of phosphorylated Mig1 was assumed to be proportional to the available amount of nuclear Mig1 (high amount of nuclear Mig1 $\rightarrow$ increased amount of shuffling Mig1 which can be phosphorylated at the phosphorylation of sites that influence Mig1 activity and localization). The minus sign represents that a phosphorylation of Mig1 reduces the amount of non-phosphorylated Mig1 in the nucleus.	k_3 Estimated from data. Varies between individuals.
$+k_4 Mig1p(t)$	k_4: Rate governing the dephosphorylation of Mig1p into Mig1. As Mig1 shuffles in and out of the nucleus (Bendrioua et al., 2014), the dephosphorylation of phosphorylation of sites in Mig1 that influence activity and localization was assumed to be proportional to available Mig1p. Here, the plus sign represents that a dephosphorylation increases the amount of non-phosphorylated Mig1 in the nucleus.	k_4 Estimated from data. Varies between cells
$-k_5\Big(1+\sigma\big(SNF1p(t)\big)\Big)Mig1(t)$	k_5: Rate governing the export of Mig1 out of the nucleus. The exact mechanism behind the nuclear export is not known, but it is known that it is dependent on SNF1p activity (Fig. 4A). Hence, it was modeled via a SNF1p-dependent sigmoid function (parametrisation in Fig. S3). As Mig1 has been shown to shuffle in and out of the nucleus regardless of glucose availability (Bendrioua et al., 2014), k_5 was used to model nuclear export for both forms of Mig1.	k_5 Estimated from data. Varies between individuals.
**Phosphorylated nuclear Mig1 (Mig1)**		
State describing the activity/amount of the phosphorylated form of nuclear Mig1.		
**Terms Mig1**	**Biological meaning and motivation**	**Notes on parameters**
$+k_3 SNF1p(t)Mig1(t)$	See above	See above
$-k_4 Mig1p(t)$	See above	See above
$-k_5\Big(1+\sigma\big(SNF1p(t)\big)\Big)Mig1p(t)$	See above	See above
**Activity of the *SUC2* promoter**		
State describing the activity of the *SUC2* promoter. More precisely, the state corresponds to the measured YFP output of the *SUC2* promoter.		
**Terms SUC2**	**Biological meaning and motivation**	**Notes on parameters**
$+\frac{k_6}{K+Mig1(t-\tau_{\mathrm{m}})}$	k_6, K: Rates governing the inhibition of *SUC2* promoter activity via SNF1pat activity. The mechanism that regulates the *SUC2* promoter is not known, hence the unidentifiable Hill-coefficient was set to the smallest value capturing the data $n = 1$. To ensure that the state corresponds to the measured YFP, the YFP maturation time was represented by a time delay τ_{m} (Fig. 3A)	k_6 estimated from data. Varies between individuals. K fixed to 0.1 to keep model identifiable τ_{m} fixed to 32 min Motivation in section.
$-k_7 SUC2(t)$	k_7: Rate governing degradation of of SUC2 promoter activity (by turnover and bleaching).	k_7 Estimated from data. Varies between individuals.
**Feedback mediating component (Y)**		
State describing the activity of the energy regulated feedback which acts on SNF1p. By the same argument as for the cascade model (Tab. S4), the feedback is modeled to be activated upon glucose drop. Furthermore, as the feedback cascade model suggested a delayed feedback, hence the response of Y was made delayed by making it dependent on the YFP activity (delayed promoter activity) of the SNF1/Mig1 regulated *SUC2*-gene. As the YFP-activity of the *SUC2* promoter depends on intracellular energy levels (e.g low activity when glucose is present), this results in Y being dependent on the intracellular energy levels. As SNF1p is regulated via several energy regulated processes (McCartney et al., 2016; Zhang et al., 2011; Ruiz et al., 2011, 2013), Y corresponds to a lumping of several cellular processes		
**Table continuous on next page**		

Terms Y	Biological meaning and motivation	Notes on parameters
$+k_8(SUC2(t)-SUC2_{t_0})$	k_8: Rate governing the production of Y via the YFP-activity of the a SNF1/Mig1 controlled *SUC2* promoter. The $SUC2_{t_0}$ was included to ensure that the feedback only is activated upon a glucose drop (see above).	k_8, Estimated from data. Varies between cells
$-k_9Y(t)$	k_9: Rate governing the degradation of feedback activity.	k_9 Estimated from data. Varies between individuals.

Table S3. Yeast strains and plasmids used in this study

Strain	Background	Description	Source
MCY60	W303	*MATa SUC2prom-Citrine-ACT1t*	Our collection
YSH202	W303	*MATa leu23/112 ura31 trp11 his311/15 ade21 can1100 GAL SUC2*	Hohmann lab
YSH372	W303	*MATa snf1*Δ*::LEU2*	Hohmann lab
YSH1545	W303	*MATa reg1*Δ*::kanMX*	Hohmann lab
YSH1832	W303	*MATa reg1*Δ*::kanMX snf1*Δ*::kanMX*	Hohmann lab
YSH2348	BY	*MATa MIG1-GFP-HIS3 NRD1-mCherry-hphNT1 MET LYS*	Hohmann lab
YSH2856	BY	*MATa MIG1-eGFP-KanMX NRD1-mCherry-HphNT1 snf1D::LEU2 MET LYS*	This study
**Plasmid name**		**Description**	**Source**
Ydp-L		*LEU2, in pRS313*	(Berben et al., 1991)
pSNF1-TAP		*URA3, in pRS316*	Hohmann lab
pSNF1-I132G-TAP		*URA3, in pRS316*	Hohmann lab

Table S4. Parameter values, standard errors (Se) and coefficient of variation (Rse) for the feedback cascade model. All fixed effects and variances (ω) have small standard errors and coefficient of variation. Some correlation parameters have large coefficient of variation, for example corr_k_7_k_6. However, in these instances the absolute uncertainty is still relatively small as the estimated parameters are close to zero. For example corr_k_7_k_6 $= 0.02$, and se_corr_k_7_k_6 = 0.0893. This suggests that the correlation, with relatively high absolute certainty, is weak for that parameter.

Parameter	Value	Se	Rse
k1_pop	0.08	0.0063	8
k2_pop	0.03	0.0024	9.5
k3_pop	5.33	0.4852	9.1
k4_pop	15.13	1.173	7.8
k5_pop	1.8	0.0171	1
k6_pop	0.73	0.0494	6.7
k7_pop	0.88	0.094	10.6
k8_pop	37.95	0.5586	1.5
k9_pop	1.74	0.27	15.5
tau_X_pop	145.09	6.452	4.4
omega_k1	0.87	0.0566	6.5
omega_k2	1.04	0.0705	6.8
omega_k3	0.98	0.0548	5.6
omega_k4	0.85	0.0535	6.3
omega_k6	0.74	0.0461	6.2
omega_k7	1.16	0.0701	6.1
omega_k9	1.71	0.0963	5.6
omega_tau_X	0.49	0.0309	6.3
corr_k2_k1	0.68	0.0511	7.6
corr_k3_k1	0.08	0.086	102.2
corr_k4_k1	-0.16	0.0895	57.5
corr_k6_k1	-0.25	0.0884	35.1
corr_k7_k1	0.11	0.0947	85.7
corr_k9_k1	-0.33	0.0795	23.8
corr_tau_X_k1	-0.29	0.0845	28.9
corr_k3_k2	-0.14	0.0841	58
corr_k4_k2	-0.45	0.0781	17.3
corr_k6_k2	-0.1	0.0971	93.3
corr_k7_k2	0.08	0.0974	116.1
corr_k9_k2	-0.31	0.081	26.1
corr_tau_X_k2	-0.07	0.0918	130.5
corr_k4_k3	0.14	0.085	62.3
corr_k6_k3	-0.06	0.0858	145.7
corr_k7_k3	0.11	0.0816	72.3
corr_k9_k3	0.64	0.043	6.8
corr_tau_X_k3	0.14	0.0822	58.1
corr_k6_k4	0.79	0.0314	4
corr_k7_k4	0.04	0.0887	213
corr_k9_k4	0.13	0.0847	67.7
corr_tau_X_k4	-0.18	0.0869	48.4
corr_k7_k6	-0.02	0.0893	543.5
corr_k9_k6	0.09	0.0861	96
corr_tau_X_k6	0.01	0.0906	608.1
corr_k9_k7	0.65	0.0485	7.5
corr_tau_X_k7	-0.23	0.0833	36.5
corr_tau_X_k9	0.13	0.0826	61.9
a	0.53	0.0036	0.7

Table S5. Parameter values, standard errors (Se) and coefficient of variation (Rse) for the feedback mediated model. All fixed effects and variances (ω) have acceptable standard errors and coefficient of variation. Some correlation parameters have large coefficient of variation, for example corr_k_4_k_2. However, in these instances the absolute uncertainty is still relatively small as the estimated parameters are close to zero. For example corr_k_4_k_2 $= 0.004$, and se_corr_k_4_k_2 = 0.11. This suggests that the correlation, with relatively high absolute certainty, is weak for that parameter.

Parameter	Value	Se	Rse
k1_pop	0.21	0.0347	16.8
k2_pop	1.11	0.0834	7.5
k3_pop	0.12	0.0181	15.1
k4_pop	3.16	0.2277	7.2
k5_pop	0.32	0.0229	7.1
k6_pop	0.41	0.0372	9.1
k7_pop	0.03	0.0025	8.3
k8_pop	0.03	0.0045	16.4
k9_pop	0.00015	1e-04	54.8
k10_pop	0.04	0.0048	12.3
omega_k1	1.84	0.109	5.9
omega_k2	0.81	0.0452	5.6
omega_k3	1.54	0.1149	7.5
omega_k4	0.61	0.0454	7.5
omega_k5	0.78	0.043	5.5
omega_k6	1	0.0552	5.5
omega_k7	0.9	0.0488	5.4
omega_k8	1.76	0.1204	6.9
omega_k9	3.87	0.4194	10.8
omega_k10	1.31	0.0886	6.7
corr_k10_k1	0.54	0.0701	13.1
corr_k2_k1	0.29	0.0811	28
corr_k3_k1	-0.41	0.0777	18.8
corr_k4_k1	-0.26	0.1033	39.1
corr_k5_k1	0.25	0.0855	33.8
corr_k6_k1	-0.29	0.0862	30.1
corr_k7_k1	-0.36	0.0785	21.7
corr_k8_k1	0.53	0.0675	12.8
corr_k9_k1	-0.26	0.0946	36.2
corr_k2_k10	0.07	0.0967	141.5
corr_k3_k10	-0.07	0.0964	129.4
corr_k4_k10	-0.26	0.1041	39.4
corr_k5_k10	0.15	0.0882	57
corr_k6_k10	-0.17	0.098	56.6
corr_k7_k10	-0.16	0.0906	58.3
corr_k8_k10	0.12	0.096	80.1
corr_k9_k10	-0.08	0.099	124.7
corr_k3_k2	0.09	0.0943	101.7
corr_k4_k2	-0.004	0.1136	2937.4
corr_k5_k2	-0.0007	0.0863	11693.2
corr_k6_k2	0.53	0.0546	10.3
corr_k7_k2	-0.29	0.0751	26.1
corr_k8_k2	0.08	0.0916	113.3
corr_k9_k2	-0.15	0.0991	67.3
corr_k4_k3	-0.03	0.1201	463.6
corr_k5_k3	-0.27	0.086	31.8
corr_k6_k3	0.22	0.0957	43.2
corr_k7_k3	-0.03	0.0904	275.3
corr_k8_k3	-0.25	0.0889	35
corr_k9_k3	0.12	0.0978	78.3
corr_k5_k4	0.31	0.0983	32
corr_k6_k4	0.07	0.1172	158.2
corr_k7_k4	0.28	0.1071	38.2
corr_k8_k4	-0.36	0.102	28
corr_k9_k4	-0.23	0.1072	46.1
corr_k6_k5	-0.3	0.079	26.2
corr_k7_k5	0.44	0.0641	14.7
corr_k8_k5	-0.16	0.0842	51.3
corr_k9_k5	-0.4	0.0762	18.9
corr_k7_k6	0.38	0.0695	18.5
corr_k8_k6	-0.28	0.0892	31.3
corr_k9_k6	-0.29	0.0929	31.5
corr_k8_k7	-0.5	0.0696	13.8
corr_k9_k7	-0.52	0.0687	13.1
corr_k9_k8	0.49	0.0786	16
a	0.53	0.0035	0.7

REFERENCES

Bendrioua, L., Smedh, M., Almquist, J., Cvijovic, M., Jirstrand, M., Goksör, M., et al. (2014). Yeast AMP-activated protein kinase monitors glucose concentration changes and absolute glucose levels. *Journal of Biological Chemistry* 289, 12863–12875. doi:10.1074/jbc.M114.547976

Berben, G., Dumont, J., Gilliquet, V., Bolle, P.-A., and Hilger, F. (1991). The YDp plasmids: A uniform set of vectors bearing versatile gene disruption cassettes forSaccharomyces cerevisiae. *Yeast* 7, 475–477. doi:10.1002/yea.320070506

Broach, J. R. (2012). Nutritional control of growth and development in yeast. *Genetics* 192, 73–105. doi:10.1534/genetics.111.135731

Hedbacker, K. and Carlson, M. (2008). SNF1/AMPK pathways in yeast. *Frontiers in Bioscience* 13, 2408. doi:10.2741/2854

Karlsson, M. O. and Savic, R. M. (2007). Diagnosing Model Diagnostics. *Clinical Pharmacology & Therapeutics* 82, 17–20. doi:10.1038/sj.clpt.6100241

McCartney, R. R., Garnar-Wortzel, L., Chandrashekarappa, D. G., and Schmidt, M. C. (2016). Activation and inhibition of Snf1 kinase activity by phosphorylation within the activation loop. *Biochimica et Biophysica Acta - Proteins and Proteomics* 1864, 1518–1528. doi:10.1016/j.bbapap.2016.08.007

McCartney, R. R. and Schmidt, M. C. (2001). Regulation of Snf1 kinase. Activation requires phosphorylation of threonine 210 by an upstream kinase as well as a distinct step mediated by the Snf4 subunit. *The Journal of biological chemistry* 276, 36460–36466. doi:10.1074/jbc.M104418200

Rubenstein, E. M., McCartney, R. R., Zhang, C., Shokat, K. M., Shirra, M. K., Arndt, K. M., et al. (2008). Access denied: Snf1 activation loop phosphorylation is controlled by availability of the phosphorylated threonine 210 to the PP1 phosphatase. *Journal of Biological Chemistry* 283, 222–230. doi:10.1074/jbc.M707957200

Ruiz, A., Xu, X., and Carlson, M. (2011). Roles of two protein phosphatases, Reg1-Glc7 and Sit4, and glycogen synthesis in regulation of SNF1 protein kinase. *Proceedings of the National Academy of Sciences of the United States of America* 108, 6349–6354. doi:10.1073/pnas.1102758108

Ruiz, A., Xu, X., and Carlson, M. (2013). Ptc1 protein phosphatase 2C contributes to glucose regulation of SNF1/AMP-activated protein kinase (AMPK) in Saccharomyces cerevisiae. *Journal of Biological Chemistry* 288, 31052–31058. doi:10.1074/jbc.M113.503763

Zhang, Y., McCartney, R. R., Chandrashekarappa, D. G., Mangat, S., and Schmidt, M. C. (2011). Reg1 protein regulates phosphorylation of all three Snf1 isoforms but preferentially associates with the Gal83 isoform. *Eukaryotic cell* 10, 1628–1636. doi:10.1128/EC.05176-11
